# Unmutated *RRAS2* emerges as a key oncogene in post-partum-associated triple negative breast cancer

**DOI:** 10.1186/s12943-024-02054-3

**Published:** 2024-07-10

**Authors:** Claudia Cifuentes, Clara L. Oeste, Isabel Fernández-Pisonero, Alejandro M. Hortal, Carmen García-Macías, Jeanne Hochart, Regina Rubira, Lydia Horndler, Carlos Horndler, Xosé R. Bustelo, Balbino Alarcón

**Affiliations:** 1 Immune System Development and Function Program, Centro Biología Molecular Severo Ochoa, Consejo Superior de Investigaciones Científicas, Universidad Autónoma de Madrid, Nicolás Cabrera 1, Madrid, 28049 Spain; 2 LynxCare, Tiensevest 132, Leuven, 3000 Belgium; 3 Centro de Investigación del Cáncer, Instituto de Biología Molecular y Celular del Cáncer, and Centro de Investigación Biomédica en Red de Cáncer (CIBERONC), CSIC-Universidad de Salamanca, Campus Unamuno s/n, Salamanca, 37007 Spain; 4 University Hospital Miguel Servet, P.º de Isabel la Católica, 1-3, Zaragoza, 50009 Spain

**Keywords:** Breast cancer, TNBC, Parity, Driver gene, *RRAS2*, RAS proteins, SNP, Genetic marker

## Abstract

**Background:**

Breast cancer (BC) is the most common cancer in women, with triple negative BC (TNBC) accounting for 20% of cases. While early detection and targeted therapies have improved overall life expectancy, TNBC remains resistant to current treatments. Although parity reduces the lifetime risk of developing BC, pregnancy increases the risk of developing TNBC for years after childbirth. Although numerous gene mutations have been associated with BC, no single gene alteration has been identified as a universal driver. *RRAS2* is a RAS-related GTPase rarely found mutated in cancer.

**Methods:**

Conditional knock-in mice were generated to overexpress wild type human *RRAS2* in mammary epithelial cells. A human sample cohort was analyzed by RT-qPCR to measure *RRAS2* transcriptional expression and to determine the frequency of both a single-nucleotide polymorphism (SNP rs8570) in the 3’UTR region of *RRAS2* and of genomic DNA amplification in tumoral and non-tumoral human BC samples.

**Results:**

Here we show that overexpression of wild-type *RRAS2* in mice is sufficient to develop TNBC in 100% of females in a pregnancy-dependent manner. In human BC, wild-type *RRAS2* is overexpressed in 68% of tumors across grade, location, and molecular type, surpassing the prevalence of any previously implicated alteration. Still, *RRAS2* overexpression is notably higher and more frequent in TNBC and young parous patients. The increased prevalence of the alternate C allele at the SNP position in tumor samples, along with frequent *RRAS2* gene amplification in both tumors and blood of BC patients, suggests a cause-and-effect relationship between *RRAS2* overexpression and breast cancer.

**Conclusions:**

Higher than normal expression of *RRAS2* not bearing activating mutations is a key driver in the majority of breast cancers, especially those of the triple-negative type and those linked to pregnancy.

**Supplementary Information:**

The online version contains supplementary material available at 10.1186/s12943-024-02054-3.

## Background

Breast cancer (BC) is the most frequently diagnosed cancer in women worldwide and is the leading cause of cancer-related deaths, the majority of which result from metastatic disease (https://www.who.int/news-room/fact-sheets/detail/breast-cancer). BC is a heterogeneous disease that is classified histologically into two main types: ductal and lobular. Invasive ductal adenocarcinomas constitute the majority (⁓80%) of newly diagnosed BCs. In pathological and molecular terms, BC is classified according to the expression of the estrogen receptor (ER), progesterone receptor (PR) and epidermal growth factor receptor 2 (HER2), and by the proportion of mitotic (Ki67^+^) cells. Accordingly, the main molecular types of BC are Luminal A, Luminal B, HER2^+^ and Triple Negative (TNBC) breast cancer. This classification has important prognostic and therapeutic implications, as it reflects the sensitivity of these tumors to hormone therapy and/or immunotherapy. TNBC is generally considered the BC type with worse outcomes due to its histological status, rate of proliferation, capacity to metastasize, and its insensitivity to hormone and antibody therapies [1].

Although childbearing is known to have a long-term protective effect against breast tumor development, studies of BC incidence in young women demonstrate a transient risk in tumor development in the years immediately following pregnancy. Compared with nulliparous women, parous women have a hazard ratio (HR) for breast cancer that peaks about 5 years after birth (HR, 1.80 [95% CI, 1.63 to 1.99]) before decreasing to 0.77 (CI, 0.67 to 0.88) after 34 years [2]. Indeed, parous pre-menopausal women have a higher incidence of BC compared to nulliparous women of the same age [3, 4]. Post-partum BC is associated with worse survival and a higher risk of metastasis than BC diagnosed in age-matched control women. This difference could be related to a higher incidence of TNBC in post-partum BC individuals [5, 6]. The transient increase in the risk of BC associated with recent pregnancy could be related to the significantly enhanced proliferation of mammary epithelial cells or post-partum/post-lactational mammary tissue involution induced by pregnancy-related hormones. Involution may mimic aspects of wound healing, including the presence of activated fibroblasts, extracellular matrix (ECM) deposition and elevated matrix metalloproteinase (MMP) levels, thus resembling a pro-tumorigenic wound environment [4].

A number of signaling pathways are particularly relevant in BC cells: the ER-signaling and Her2-signaling pathways for ER^+^ and HER2^+^ tumors, as well as the Wnt/β-catenin and PI3K/Akt/mTOR pathways. Indeed, alterations to genes that participate in these pathways have been detected in BC [7]. Germline mutations in the *BRCA1*, *BRCA2*, *PTEN* or *TP53* tumor suppressor genes indicate that 5–10% of BCs are familial. These genes may accumulate somatic mutations in conjunction with mutations and/or amplifications of PIK3CA and AKT3, as well as deletions or mutations of PTEN, TSC1, and INPP4B in the PI3K/mTOR pathway, although not in the majority of breast cancers (BCs). In addition, *FGFR1*, *EGFR*, *IGF1R*, *ERBB2*, *ERBB3*, *ERBB4* and *BRAF* may be amplified in BC, or carry activating mutations. However, none of them have been directly established as a sole driver gene capable of inducing the entire process of transforming epithelial cells into breast cancer.

Regarding the classic RAS family members, and unlike pancreatic, lung and colorectal cancers, activating oncogenic mutations in *KRAS*, *NRAS* and *HRAS* have not often been found in BC [8]. Indeed, the frequency of mutation of these three genes together is lower than 1% in genome-wide association studies (GWAS) of BC (www.cbioportal.org). Nonetheless, wild-type (WT) *HRAS* and *KRAS* seem to play an important role in the progression, dissemination and resistance to therapy in BC [8]. For wild type *NRAS* it has been shown that its overexpression enhances invasiveness of basal-like breast cancer cells, but a driver role of wild type *NRAS* has not been demonstrated [9]. The *RRAS2* member of the RAS-related subfamily, also known as TC21, is a close relative of the classic RAS GTPases. While this gene is infrequently mutated in BC [10], which was corroborated by GWAS, overexpression of the wild type form of *RRAS2* induces the transformation of a BC cell line [11]. Conversely, analysis of a *Rras2* null mouse mutant showed that this GTPase is necessary for correct mammary gland development [12]. In addition, reducing R-RAS2 expression in human BC cell lines through knock-down strategies revealed that it is necessary for tumor growth following orthotopic transplantation into mice, and for late-stage metastasis to the lungs and/or spleen [13]. Furthermore, the effects of R-RAS2 expression were linked to the activation of the PI3K/Akt pathway by this GTPase, independently of the mutational status of *KRAS*. Moreover, a polymorphism in the *RRAS2* promoter is associated with an unfavorable tamoxifen treatment outcome [14] and, conversely, silencing the *RRAS2* gene enhances the tamoxifen sensitivity of a BC cell line [15]. Those data indicate that *RRAS2* is an important gene in the development and clinical outcome of BC.

Previous studies in mice established R-RAS2 as an important player in immunological development and homeostasis. R-RAS2 binds to antigen receptors on B and T cells (the BCR and TCR, respectively) through their immune Receptor Tyrosine Activation Motifs (ITAMs), and it mediates tonic signaling from these key hubs, mainly through PI3K pathways [16]. Downstream of PI3K, R-RAS2 can propagate signals intracellularly through Akt and NFκB [17]. In B cells, we previously showed that, as an effector of the BCR, R-RAS2 is required for an efficient germinal center reaction by regulating B cell metabolism [18]. To determine if unmutated *RRAS2* overexpression drives the generation of lymphoid cancers, we generated a knock-in mouse line at the Rosa26 locus. All these mice developed chronic lymphocytic leukemia (CLL), a B-cell leukemia, reflecting the driver role of this RAS GTPase in the absence of activating mutations [19]. Moreover, we found that a single nucleotide polymorphism in the 3’-untranslated region (UTR) of the *RRAS2* mRNA (rs8570) was associated with stronger overexpression of *RRAS2* in human CLL.

Here we show that, in addition to CLL, female mice overexpressing *RRAS2* in all tissues develop breast cancer, but only after going through pregnancy. BC development was also detected in all female mice in a pregnancy-associated manner, when *RRAS2* overexpression was restricted to the mammary tissue, suggesting it is tissue-intrinsic. The driver role of *RRAS2* overexpression in mice is paralleled by the finding of overexpression of *RRAS2* in more than two thirds of patients’ samples, of which, those from young parous women and of TNBC type are the ones with highest expression of *RRAS2*. Finally, we found an overrepresentation of tumors with homozygosity for the alternate C allele at SNP rs8570 position and an increased number of copies of the *RRAS2* gene in both tumoral and healthy tissue of BC patients. These genetic data strongly suggest overexpression of *RRAS2* is causative of human breast cancer.

## Materials and methods

### Mice

The Rosa26-*RRAS2*^*fl/fl*^ knock-in mouse line was generated by homologous recombination as described [19], and it contains the WT human *RRAS2* sequence with an HA-tag driven by a CAG promoter, followed by an EGFP sequence downstream of an IRES sequence, and with a LoxP-flanked stop codon at the 5’ end of the construct (Rosa26-*RRAS2*^*fl*/fl^). Rosa26-*RRAS2*^*fl/fl*^ mice were crossed with Cre recombinase lines to induce conditional overexpression of this construct by removing the stop codon. We first studied systemic *RRAS2* overexpression using the Sox2-Cre transgenic mice kindly provided by Dr César Cobaleda (Centro de Biología Molecular Severo Ochoa-CBMSO, Madrid). As Sox2 is an embryonic stem cell transcription factor, the LoxP-flanked sequence is deleted in all tissues in these mice. By crossing with the Wap-Cre and MMTV-Cre lines purchased from the NCI Mouse Repository (Frederick, MD), we generated mammary epithelium specific *RRAS2*-overexpressing mouse lines. In the Rosa26-*RRAS2*^*fl/fl*^ x Wap-Cre strain, the Cre recombinase is expressed under the control of the Wap (whey acidic protein) promoter, specifically in the secretory epithelium of the mammary gland during first pregnancy. Rosa26-*RRAS2*^fl/fl^ x MMTV-Cre mice overexpress *RRAS2* in virgin and lactating mammary gland, and Cre recombinase has also been detected in other tissues [20]. All the mice were maintained under SPF conditions at the animal facility of the CBMSO, in strict accordance with national and European guidelines. All the procedures were approved by the ethical committee at the CBMSO and by the Regional Government of Madrid (authorization numbers PROEX 384/15 and PROEX 296.7/21).

### Human tissue samples

Samples and data from the patients included in this study were provided by Biobanks integrated into the Spanish National Biobanks Network, including the Aragon Health System Biobank (AHSB) (PT17/0015/0039) and the Fundación Instituto Valenciano de Oncología (FIVO) Biobank (PT17/0015/0051). Samples were processed following standard operating procedures with the approval of the appropriate Ethics and Scientific Committees. In total, 200 blood samples and 449 tumor samples from breast cancer patients were analyzed. A cohort of 234 human capillary blood samples was obtained from healthy volunteers from CBMSO who had provided their informed consent for genomic DNA extraction and MARBiobanc from IMIM (Institut Hospital del Mar d’Investigacions Mèdiques, Barcelona, Spain) (ISO 9001:2015). The study was carried out in compliance with EU guidelines and following the ethical principles of the Helsinki Declaration. All sample donors were of Caucasian race.

### Immunohistochemistry and immunocytochemistry

Extracted organs and tumors were fixed in 4% PFA (paraformaldehyde) at 4 °C O/N (overnight). Subsequent processing for paraffin block preparation and sectioning was carried out at the Histology Facility of CNB (Spanish National Centre for Biotechnology, Madrid, Spain), as well as hematoxylin–eosin staining and Masson’s trichrome stain. For immunohistochemical staining, two incubations in xylene for 5 min each were conducted, followed by two times in 100%, 95%, 75%, and 50% ethanol, each for 5 min. Subsequently, samples were treated with methanol + 0.3% H_2_O_2_ for 20 min and washed twice for 5 min with distilled water. Antigen retrieval was carried out by incubating with 1 × citrate buffer (Sodium Citrate 1 × pH 6.0, 0.1 M Citric acid) at boiling temperature for 10 min, followed by two washes with PBS (Phosphate-buffered Saline) for 5 min each after allowing the samples to cool down. Tissue sections were then blocked by incubating with PBS + 1% BSA (Bovine Seroalbumin) at 4 °C for 30 min. Staining with the corresponding primary antibodies was performed in PBS + 3% BSA at 4 °C O/N. The primary antibodies used for immunohistochemistry were Erbb2 (#2165, Cell Signaling, 1:200); PR (Polyclonal, Proteintech, 25,871–1-AP 1:200); ERα (Clon E115, Abcam, ab32063 1:500), HA influenza hemagglutinin epitope (12CA5, Sigma, 1:500) and Ki-67 proliferation marker (Polyclonal, concentration of use 5 µg/ml). Next, incubation with the corresponding secondary antibody was carried out at 4 °C for 30 min (Polyclonal Biotinylated anti-Rabbit, DAKO E0432, 1:200 and Polyclonal Biotinylated anti-Mouse, Vector Laboratories BA-2000, 1:200). Detection of the antigen–antibody complexes was carried out using the DAB chromogen (3,3-Diaminobenzidine), incubating all the staining samples for the same time. A counterstaining with hematoxylin was performed to stain the nuclei for 10 s, followed by washes in water to remove excess, and rinsing with PBS. Samples were dehydrated by sequential incubations with distilled water for 5 min, followed by two washes of 50%, 75%, 95%, and 100% ethanol for 2 min each, and three 3-min washes with xylene. Samples were mounted using DPX mounting medium. Microscopy was performed using a Vertical microscope AxioImager M1 (Zeiss) coupled with DMC6200 camera (Leica) and LAS X software (Leica).

### Whole mount staining and mammary structures counting

The mammary glands from the left fourth pair were extracted from female mice for whole- mount staining with Carmine Alum. The isolated mammary glands were air dried for 10–15 min on a clean glass slide, followed by fixation in Carnoy’s solution (60% ethanol, 30% chloroform, 10% glacial acetic acid) for 4 h at RT (room temperature). The slides were sequentially washed in 70%, 35% and 15% ethanol for 15 min each, and rinsed in distilled water for 5 min. Samples were stained RT O/N with Carmine Alum dye, followed by sequential dehydration in 70%, 95% and 100% ethanol. Glands were cleared in xylene as required, mounted with Permount mounting media (Fisher Scientific), and covered with coverslips.

Images of the whole mounts were acquired using an Olympus digital camera under constant lighting conditions and with a Vertical microscope AxioImager M1 coupled with a DMC6200 camera and equipped with LAS X software. According to defined guidelines, Terminal End Buds (TEBs) were identified as protrusions located at the end of ducts. Alveolar buds were recognized as small, round structures that were not yet organized into lobules. Each structure was classified as one single alveolar bud. Alveoli were observed as discrete units, with each lobule counted as one unit [21]. The experiment was conducted analyzing three independent animals with a minimum of four mammary glands per group and development stage. Four randomly chosen regions of 4 mm^2^ per mammary gland were selected for counting. The number of structures was manually counted using ImageJ software (Wayne Rasband, National Institutes of Health, Bethesda, Maryland, USA) under magnification, plotting the total number of observed structures per section.

### RNA extraction

Mouse breast tumors were isolated and passed through 40 μm Cell Strainers (BD Pharmingen); healthy mammary gland tissue was finely minced using a 22 scalpel. The minced tissue was then digested in DMEM supplemented with 5% FBS and 2 mg/ml Collagenase from Clostridium histolyticum (Sigma) for 1 h at 37ºC under moderate shaking. To separate mammary organoids from fat, fibroblasts, and red cells, a series of centrifugation steps were performed at 600 g for 10 min, 5 min, and 4 rounds of 2 s each.

Human breast cancer samples were supplied in CNB (core needle biopsy) and OCT (optimal cutting temperature compound) format. CNB tissue was homogenized with RNAse free Lysing Matrix Tubes in a MagNa Lyser Rotor (Roche Instrumentation). OCT samples were homogenized using 20 G needles.

TRIzol (Invitrogen) was added to the different homogenates. Subsequently, chloroform (Merck) was added, and the mixture was vigorously vortexed. After a 15-min centrifugation at 12,000 g, the aqueous phase was carefully transferred to another tube and cold isopropanol (Merck) was added and incubated for 10 min at RT. After another centrifugation at 12,000 g for 10 min, the pellet was washed with 70% ethanol and centrifuged for 5 min at 10,000 g. The ultimate pellet was diluted in RNAse-free water and stored at -80 °C for preservation.

### gDNA extraction

Tissue homogenates and cell line pellets were resuspended in 500 μL of Lysis Buffer (50 mM Tris–HCl ph = 8.0, 200 mM NaCl, 10 mM EDTA, 1% SDS) containing 0.2 mg/ml Proteinase K (Sigma) and incubated O/N at 55ºC. For mouse genotyping, gDNA was extracted from a small fragment of the tail cut when the animal was 3- 4 weeks old. In the case of peripheral blood, red blood cells were eliminated by incubating the samples with 1 ml of ACK Lysis Buffer (0.15 M NH_4_Cl, 10 mM KHCO_3_, 0.1 mM EDTA, pH 7.2- 7.4) for 5 min at RT. Then, samples were centrifuged at 1,500 g for 5 min and the remaining cells were resuspended in Lysis Buffer with Proteinase K as described. On the following day, 500 μL Phenol–Chloroform (Sigma) was added to each sample, and then centrifuged at 9,500 g at RT for 10 min. Chloroform was added to the aqueous phase and samples were centrifuged again at 9,500 g at RT for another 10 min. Afterwards, 1 mL of absolute ethanol along with 1 μL Pellet Paint (Merck) were added to the samples. Then, they were centrifuged at 9,500 g at 4ºC for 10 min. The resulting pellet was washed with 70% ethanol and the samples were once again centrifuged at 9,500 g and 4ºC for 10 min. Finally, gDNA was eluted in 8 mM NaOH, and the pH was adjusted with HEPES. After an O/N incubation at 4ºC, all gDNA samples were sonicated before measuring their concentration.

### Real-time PCR

From the total RNA isolated, cDNA was synthesized with NZY First Strand cDNA Synthesis kit (NZYtech) along with Oligo-dT primers. Quantitative Real-Time Polymerase Chain Reaction (RT-qPCR) was then performed in triplicate using 50 ng cDNA per well as template. The reaction was performed with the NZYSupreme qPCR Green Master Mix (NzyTech) and gene- specific primers in a CFX384 Touch Real-Time PCR Detection System (BioRad). All oligonucleotide sequences are provided in their 5’-3’ orientation. A set of primers was used to measure mRNA expression of human *RRAS2* exclusively or a combination of human and mouse *RRAS2*/*Rras2.* (human *RRAS2*: GCAGGACAAGAAGAGTTTGGA and TCATTGGGAACTCATCACGA; human and mouse *RRAS2*/*Rras2*: GAGTTTGGAGCCATGAGAGA and CCTTTACTCTGAGAATCTGTCTTTGA).

For mouse samples, the normalizer gene used was C-Terminal Binding Protein 1 (*Ctbp1;* GTGCCCTGATGTACCATACCA, GCCAATTCGGACGATGATTCTA), as it has been validated as a reliable, tissue- specific reference gene for mouse mammary gland studies [22]. For human samples, the normalizer gene was Pumilio Homolog 1 (*PUM1,* AGTGGGGGACTAGGCGTTAG, GTTTTCATCACTGTCTGCATCC). *PUM1* has been previously recognized as one of the most stable reference genes for characterizing human tumors [23]. Each plate used in the RT-qPCR assays for human BC samples included a consistent set of 10 tumor-adjacent normal tissue samples to ensure precise normalization.

Quantitative PCR has also been widely utilized for quantification of copy number variation. The analysis involves amplification of a test locus with unknown copy number and a reference locus with known copy number. This method has been used for example for the determination of HER-2/neu amplification in human breast carcinoma and has been defined as useful alternative to FISH in breast cancer patients [24, 25]. We characterized amplifications in the *RRAS2* gene in human breast cancer samples specifically targeting the exon 2 region of *RRAS2* (CGGGCTGCTCTGTCATCTATC, CCATGCCTGGCCATGAATTTTA) in order to prevent any non-specific amplification of RAS genes due to their substantial sequence similarity. For normalization, *COX8A* (ATCATGTCCGTCCTGACGCC, CCGTTCCTCACCATGATCCC) was employed.

### RNAseq of mouse tumors and differential gene expression analysis

Six breast tumor samples from Rosa26-*RRAS2*^fl/fl^ x Wap-Cre mice, five samples from Rosa26-*RRAS2*^*fl*/fl^ x MMTV-Cre mice and four samples from Rosa26-*RRAS2*^fl/fl^ x Sox2-Cre mice were sequenced, along with normal mammary gland samples (2 wild-type nulliparous, 3 wild-type breeders, 2 Cre- Rosa26-*RRAS2*^fl/fl^ nulliparous females and 3 Cre- Rosa26-*RRAS2*^fl/fl^ breeder mice). The quality control, library preparation and data processing were carried out at the Centre for Genomic Regulation (CRG, Barcelona, Spain). Samples were sequenced with 50 base pair single-end read using a HiSeq 2000 platform (Illumina) after quality control of the samples on a Bioanalyzer Instrument (Agilent). The quality of sequencing data was assessed using FastQC software. The generated libraries were then aligned to the genome reference sequence of *Mus musculus* GRCm39 from Ensembl. The alignment was performed using the Spliced Transcripts Alignment to a Reference (STAR) software. Read counting was carried out with the FeatureCounts tool, and differential gene expression analysis between sample groups was performed in an R environment (version 4.2.2.) with Bioconductor using the DESeq2 package (V1.38.2) [26]. For differential gene expression analysis, the “apeglm” method was used to obtain unbiased logFC estimates, specifically targeting accurate identification of significant differences in the dataset. Genes with adjusted *p*-values < 0.05 after Benjamini–Hochberg correction were identified as differentially expressed genes. Fast Gene Set Enrichment Analysis was performed with the fgsea package (V1.2.4.0). Ranked gene lists derived from DESeq2 test statistics were compared to predefined gene sets from the MSigDB database [27] of Gene Ontology, Hallmarks, Reactome and community contributors. For the analysis, 1000 permutations were utilised.

### Integration of mouse and human RNAseq data and clinical traits from TCGA dataset

The Cancer Genome Atlas (TCGA) consortium has collected tumors from 161 tissue source sites across the world, acquiring tumors from 11,160 patients with 33 different cancer types [28]. In this study, the Breast Invasive Carcinoma dataset was studied, including 1,116 samples of breast invasive carcinomas as well as 112 samples of normal mammary tissue. Raw counts of bulk RNAseq data, coupled with the associated clinical attributes, were obtained with the GDCquery function found in the TCGAbiolinks package, integrated within the R/Bioconductor environment. Count data from 1,228 samples were filtered to remove lowly-expressed genes. For Principal Component Analysis, the data underwent preprocessing via variance stabilization using the vsd function to normalize gene expression data, in combination with *RRAS2*-overexpressing mouse breast tumors RNAseq data. Raw count data from mouse breast tumors developed under *RRAS2* overexpression and from breast cancer patients were merged by combining human and murine gene annotations using the BioMart tool from Ensembl (https://academic.oup.com/nar/article/51/D1/D933/6786199?login=true). Subsequently, counts were filtered to remove lowly-expressed genes and transformed for normalization using the vst() function, in order to stabilize count data variances. Principal Component Analysis (PCA) was then conducted using the pca() function. For this analysis, samples were classified according to their murine or human origin and, in the latter case, according to their previously defined molecular subtype. In addition, hierarchical clustering was performed. For this cluster analysis, genes were sorted by variance, selecting those above the cutoff value of 0.995, representing the top 0.5% of genes with the highest variances. A total of seventy genes met these criteria. To build the heatmap, 80 random samples from each subtype of breast cancer patients were selected, along with the 15 mouse breast tumors. The heatmap was generated using the pheatmap() function using “ward.D2” as hierarchical clustering method.

### Triple-negative breast cancer molecular subtyping

By combining microarray expression data from 21 breast cancer datasets, which included 587 TNBC cases, Lehmann et al. identified by cluster analysis six TNBC subtypes with unique gene expression profiles: two basal-like (BL1 and BL2), an immunomodulatory (IM), a mesenchymal (M), a mesenchymal stem-like (MSL), and a luminal androgen receptor (LAR) subtype [29]. We analyzed the gene expression data from our mouse breast tumors generated under wild-type *RRAS2* overexpression to determine which of the defined subtypes they resemble. For this, we used the web-based subtyping tool TNBCtype, which was developed for the characterization of TNBC samples based on Lehmann’s gene expression data and classification methods [30]. This analysis allowed us to obtain the corresponding correlation coefficient and permutation P-value for each TNBC subtype.

### Western blot assays

Mouse mammary tumors and normal mammary tissue were minced, digested, and processed to separate mammary organoids as previously described. Cell aggregates were resuspended in RIPA lysis buffer (10mM Tris–HCl, pH 8.0, 1mM EDTA, 0.5mM EGTA, 1% Triton X-100, 0.1% Sodium Deoxycholate, 0.1% SDS, 140mM NaCl) containing protease and phosphatase inhibitors, mechanically disaggregated using a Polytron® tissue homogenizer, and lysed for 2 h under rotation at 4°C. Cell lysates were separated by SDS-PAGE and transferred to nitrocellulose membranes. Membranes were then blocked for 1 h in TBS-Tween with 5% BSA and incubated overnight at 4°C with primary antibodies diluted in blocking buffer. The primary antibodies used included anti-phospho-AKT S473 (#4060), phospho-FoxO1 (Thr24)/FoxO3a (Thr32) (#9464), phospho-MEK1/2 (Ser217/221) (#9121), phospho-p44/42 MAPK (ERK1/2) (Thr202/Tyr204) (#4370), phospho-S6 Ribosomal Protein (Ser240/244) (#2217), and β-Actin (#4967) from Cell Signaling, and anti-HA from Sigma. Primary antibodies were used at 1:1000 dilution, except β-Actin at 1:20,000. The next day, membranes were washed twice with TBS-Tween and incubated for 45 min at RT with secondary antibody (1:30,000, Jackson Immunoresearch). After at least four washes, antibody binding was detected using standard chemiluminescence with a Kodak X-OMAT 2000 Processor.

### Subcutaneous administration of β-estradiol and progesterone

To study the effects of β-estradiol and progesterone on *Rras2* levels in the mammary gland, 12-week-old wild-type C57BL6/J females were housed for 14 days prior to hormone administration to synchronize their estrous cycles by the Lee-Boot effect [31]. To confirm estrous cycle synchronization, vaginal cytological evaluation was conducted through vaginal lavage followed by crystal violet staining [32]. Mice were administered subcutaneous 17β-estradiol (1 μg in sesame oil) and progesterone (1 mg in sesame oil) or control sesame oil daily for 5 days. This hormone injection protocol has previously been shown to promote mammary gland development [33, 34]. Subsequently, the females were sacrificed, and mammary glands were isolated and processed for RNA extraction as previously described, followed by RT-qPCR assays to assess *Rras2* levels.

### Luciferase assays

For luciferase assays, the pGL3-Control Firefly Luciferase Vector was utilized to clone either the canonical 3’-UTR of *RRAS2* mRNA or the 3’-UTR containing the rs8570 SNP. Simultaneously, the intrinsic SV40 polyadenylation signal within the vector was removed through Gibson Assembly. The pGL3-RRAS2 3’-UTR WT or G124C were transiently co-transfected with Renilla Luciferase pRL-SV40 vector into CAL-51 cells using the jetPEI reagent (Polyplus) in p24-well plates. After 48 h, luciferase levels were assessed through the Dual- Luciferase® Reporter Assay System (Promega). Cell lysates were prepared utilizing 100 μL of Passive Lysis Buffer from the Dual-Luciferase Reporter Assay System. Non transfected cells were used as negative control, in order to determine the background signal from the measurement of total luminescence. Then, 20 μL of cell extracts were dispensed per well of a p96-well plate and loaded into a FLUostar Optima microplate reader equipped with syringe injectors. In addition to quantifying the luminescence of the luciferase signals, the stability of the mRNA of the *luc* gene, containing the different 3’UTR regions within the different BC cell lines, was quantified by RT-qPCR.

### Sequencing strategy for patient samples

The technology used for the characterization of the rs8570 SNP involves a Fluorescent dual-labelled probe system, which was pre-developed by Applied Biosystems (Foster City, CA, USA). In summary, the TaqMan® MGB (minor groove binder) probes consist of specific oligonucleotides for the target SNP that carry a reporter VIC™ fluorescent dye at the 5 ´ end of the probe for allele 1 (124G) or a reporter FAM™ fluorescent dye for allele 2 (124C). Both probes are equipped with a non-fluorescent quencher (NFQ) dye at their 3 ´ ends. The minor groove binder stabilises the target-probe duplex. During the PCR reaction, the AmpliTaq Gold™ DNA polymerase used in the assay specifically cleaves the probes that are 100% complementary to the DNA template. This will lead to the release of the fluorescent reporters and fluorescence emission through the removal of the NFQ. Reactions were performed with 20 ng of template and an initial cycle of 10 min at 95ºC followed by 40 cycles of 15 s at 95ºC and 1 min at 60ºC.

When analyzing paired samples of tumor tissue and adjacent healthy margins, due to the limited availability of non-tumoral tissue, we implemented an alternative strategy. In this subset of samples, RNA was extracted and the characterization of the rs8570 SNP in RRAS2 3’UTR was achieved through nested PCR utilizing cDNA, as detailed in previous studies [19]. For the nested PCR, two reactions were conducted, each with 30 cycles of 45 s at 95ºC, 45ºC at 60ºC and 2 min at 72ºC. The oligonucleotides used for each PCR reaction are GCAGGACAAGAAGAGTTTGGA and TGAAGCAGCCTTAGTGTTTCCTT for the first reaction, TCCATGAACTTGTCCGGGTT and TGAAGCAGCCTTAGTGTTTCCTT for the second PCR. This PCR product was sent to sequence by the Sanger method (Eurofins Genomics).

### Statistical analysis

Statistical parameters, including the exact *n* value, and the mean ± S.D. or ± S.E.M. are described in the figures and figure legends. Non-parametric Wilcoxon, Mann–Whitney, Kolmogorov–Smirnov tests, and parametric Student’s T-tests and One or Two-way ANOVA tests were used to assess the significance of the mean differences or cumulative distributions as indicated. All the data were analyzed using the GraphPad Prism 9.5.1 software. Experiments were independently repeated as described in the figure legends. The number of mice used for comparison was calculated with the aim of generating significant data to give an alpha = 0.05 and a standard deviation of about 0.3 when statistical tests were conducted with the minimum number of animals. The different deviation of the control and test groups suggested the use of different numbers of each animal for the definitive experiments.

## Results

### Wild type RRAS2 is an oncogenic driver in breast cancer when overexpressed

**Fig. 1:**
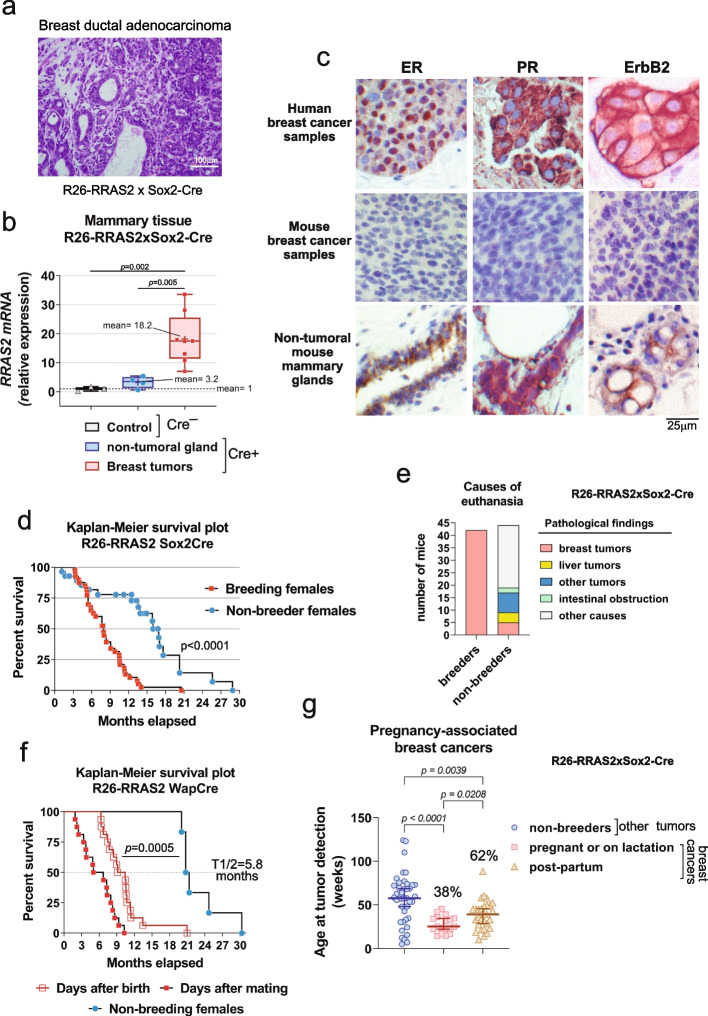
Overexpression of wild-type *RRAS2* drives the generation of pregnancy-related breast cancers. **a** Hematoxylin and eosin staining of a paraffin section from a tumor emerging in the left axillary mammary gland of a 5-month-old Rosa26-RRAS2^fl/fl^ x Sox2-Cre female breeding mouse. Scale bars indicate 100 μm. **b** Box and whisker plot showing all the points and the median value of RT-qPCR data showing *RRAS2* mRNA expression in non-tumoral and tumoral mammary tissues from Rosa26-RRAS2^fl/fl^ x Sox2-Cre female breeding mice. The expression data is normalized to the mean value of non-tumoral mammary tissue from Cre^–^ RRAS2^fl/fl^ female breeding mice (*n* = 3) and significance was assessed with a one-way ANOVA using Dunnett’s multiple comparisons test. **c** Immunoperoxidase staining of tissue paraffin sections from Rosa26-*RRAS2*^fl/fl^ x Wap-Cre female mice showing estrogen receptor (ER), progesterone receptor (PR) and ErbB2 expression in the non-tumoral and tumoral ductal epithelial cells of the same individual mice. In parallel, paraffin sections of human breast cancer positive for ERα, PR, or ERBB2 were stained with the same antibodies and used as positive controls. ERα staining is clearly concentrated in the nuclei of the positive cells, ErbB2 staining produced a plasma membrane staining, and PR staining resulted in a mixed, mostly cytoplasmic pattern. Scale bar indicate 25 μm. **d** Kaplan–Meier survival plot of Rosa26-*RRAS2*^fl/fl^ x Sox2-Cre female breeding (*n* = 32) and non-breeding mice (*n* = 24) allowed to age in the same housing conditions. The median survival for female breeders was 8.2 months and the median survival for non-breeding female controls was 17 months, assessing significance with a log-rank Mantel-Cox test. **e** Classification of Rosa26-*RRAS2*^fl/fl^ x Sox2-Cre female mice of panel F according to the causes of euthanasia found after necropsy. **f** Kaplan–Meier survival plot of Rosa26-*RRAS2*^fl/fl^ x Wap-Cre female breeding (*n* = 16) and non-breeding mice (*n* = 6) allowed to age in the same housing conditions. Survival data for breeders is provided as months of age and as months after having the first litter The median survival (T_1/2_) for female breeders was 9.8 months of age and 5.8 months after giving birth. and the median survival for non-breeding female controls was 21 months, assessing significance with a log-rank Mantel-Cox test. **g** Age at tumor detection in non-breeder Rosa26-*RRAS2*^fl/fl^ x Sox2-Cre female mice (tumors other than breast cancer; *n* = 41) and Rosa26-*RRAS2*^fl/fl^ x Sox2-Cre breeder female mice, classified the latter according to the breast cancers being found during pregnancy or lactation (*n* = 17) or post-weaning (*n* = 29). Significance was assessed using a one-way ANOVA Dunnett’s multiple comparisons test

Previously, we described the generation of a line of mice genetically modified to overexpress wild-type human *RRAS2* in all tissues (Rosa26-*RRAS2*^fl/fl^ x Sox2-Cre) [19]. We now found that female mice of that line developed tumors at any of the 5 mammary gland sites, categorized as breast ductal adenocarcinomas upon histopathological analysis (Fig. 1a). In the non-tumoral mammary glands of these mice, *RRAS2* mRNA was expressed 3.2-fold more strongly than in Sox2-Cre^—^ control mice, and a mean of 18.2-fold higher in the tumor tissue, suggesting a process of progressive upregulation of *RRAS2* mRNA expression after the initiation event in mammary glands of knock-in mice (Fig. 1b). In order to address if *RRAS2* overexpression induced BC in a breast-intrinsic manner, we crossed Rosa26-RRAS2^fl/fl^ mice with mouse mammary tumor virus (MMTV)-Cre and with Wap-Cre mice. Although not ubiquitous, the MMTV promoter is active in tissues other than the mammary gland epithelium; the whey acidic promoter (Wap) is however expressed in breast during pregnancy [35–37]. All breeder females developed breast ductal adenocarcinomas and had median survival times close to those of Sox2-Cre^+^ mice (Supplemental Fig. S1a), thus confirming that the effect of *RRAS2* overexpression is breast epithelium-intrinsic.

When compared with tissue sections of human Luminal A and HER2 + breast cancers, immunohistochemical analysis of breast ductal adenocarcinomas from Rosa26-*RRAS2*^fl/fl^ x Wap-Cre mice revealed the lack of ERα, PR, and ErbB2 markers, (Fig. 1c). Likewise, the comparison with murine normal tissues known to express ERα, PR or ErbB2, showed again that the breast tumors developing in Rosa26-*RRAS2*^fl/fl^ x Wap-Cre mice were negative for the three markers (Fig. S1b). Thus, these results showed that the breast tumors developing in mice overexpressing *RRAS2* resemble the human Triple Negative Breast Cancer tumors (TNBC). Surprisingly, we found that the Rosa26-*RRAS2*^fl/fl^ x Sox2-Cre female mice, with ubiquitous expression of *RRAS2*, that developed breast cancer (BC) were only those that had been pregnant (breeders). Indeed, comparing the survival curves of breeder female mice with that of non-breeder females, we found that the median survival for the former was 8 months as opposed to a median survival for the latter of 17 months (Fig. 1d). All breeder female mice had to be sacrificed as they developed BC, whereas most (⁓80%) non-breeder females were sacrificed later due to the development of other types of tumors, including CLL and liver carcinomas (Fig. 1e). Rosa26-*RRAS2*^fl/fl^ x Wap-Cre female mice all developed BC when in crossing and had median survival times close to those of Sox2-Cre + mice (Fig. 1f). The association between *RRAS2-*induced breast cancer and pregnancy was also manifested by the fact that 38% of tumors were detected in Rosa26-*RRAS2*^fl/fl^ x Sox2-Cre female mice that were either pregnant or breast-feeding and were significantly younger than female breeders whose tumors were detected weeks after weaning (post-partum, Fig. 1g). All these data demonstrate that overexpression of wild-type *RRAS2* in mice causes breast cancer in a pregnancy-related manner.

### *RRAS2* overexpression causes hyperproliferation and alterations of the ductal epithelium both during pregnancy and postpartum breast involution

**Fig. 2:**
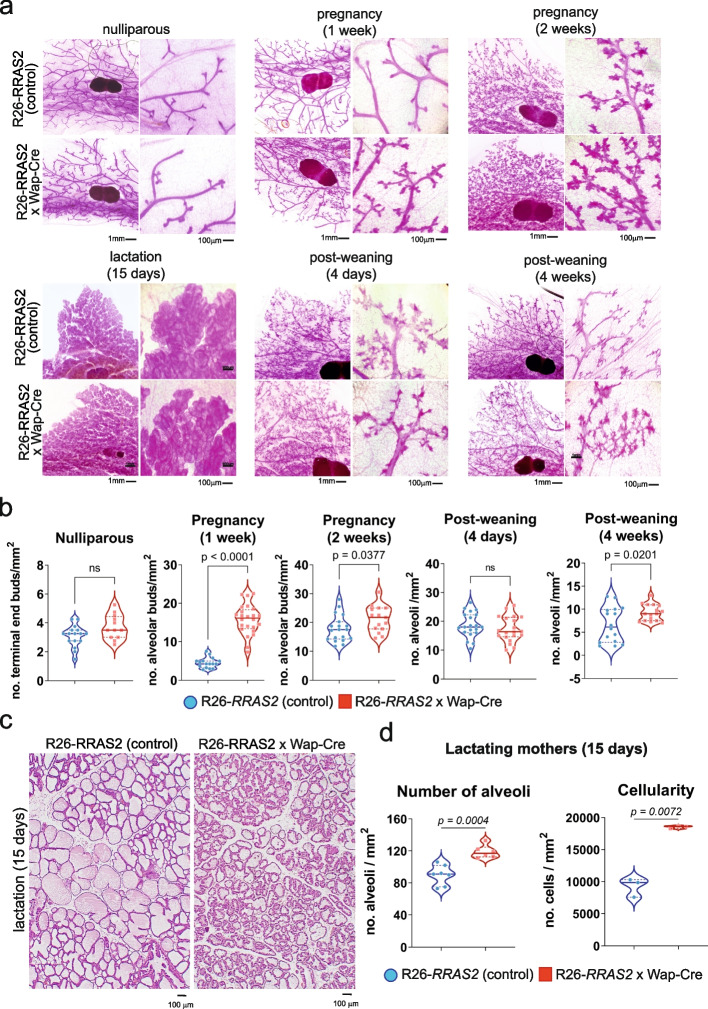
Overexpression of wild-type *RRAS2* causes overproliferation of alveoli and ductal cells during pregnancy and post-weaning. **a** Carmine alum stainings of the 4th pair mammary gland from the right flank of control Rosa26-*RRAS2*^fl/fl^ (Cre^—^) and Rosa26-*RRAS2*^fl/fl^ x Wap-Cre females taken at different stages: 8-week old nulliparous mice, at 1 week and 2 weeks of pregnancy, at day 4 after ceasing lactation (post-weaning), and at 4 weeks post-weaning. Two representative images at 1 × and 5 × are shown per condition. Scale bars indicate 1 mm and 100 μm, respectively. **b** Violin plots showing all data points, the median value and quartiles of the number of structures quantified in four independent 2 × 2 mm microscopy fields per mammary gland. A minimum of four glands from 3 animals per condition were considered for analysis. Significance was assessed using two-tailed unpaired t-tests. **c** Hematoxylin and eosin stainings of the right 4th pair mammary gland of control and Rosa26-*RRAS2*^*fl*/fl^ x Wap-Cre females at day 15 of lactation. A representative image at 5 × per condition is shown. Scale bars indicate 100 μm. **d** Violin plots show all data points, the median value and quartiles of the number of alveoli and cells in four independent 2.0 × 1.3 mm microscopy fields per mammary gland photographed at a 5 × magnification. A minimum of four glands from 3 animals per condition were considered for analysis. Significance was assessed using two-tailed unpaired t-tests

We have previously found in *Rras2*-deficient mice that this GTPase is required for branching and remodeling of the mammary gland during puberty [12], we hypothesized that *RRAS2* overexpression could lead to alterations in these processes. To test this hypothesis, we carried out a series of carmine alum stainings of complete mammary glands of female Rosa26-*RRAS2*^fl/fl^ x Wap-Cre mice at different stages of their reproductive life. We found no differences with control mice in mammary glands of adult nulliparous mice in terms of branching or density of terminal-end buds (TEBs, Fig. 2a and b). This uniformity changed with pregnancy: the *RRAS2*-overexpressing Rosa26-*RRAS2*^fl/fl^ x Wap-Cre mice quadruplicated the median and triplicated the mean of alveolar buds at 1 week of pregnancy (Fig. 2b). Such differences diminished at the 2nd week of pregnancy, although still remained statistically higher in Rosa26-*RRAS2*^fl/fl^ x Wap-Cre mice. During lactation, the milk-filled ducts of both groups of mice were not found to be grossly different by carmine alum staining (Fig. 2a). However, after weaning, *RRAS2*-overexpressing mice reduced the number of alveoli more slowly than control mice (Fig. 2b, 4-weeks vs 4 days post-weaning). The overabundance of alveoli in RRAS2-overexpressing mice was also evident in hematoxylin and eosin stainings of mammary glands of lactating mothers at day 15 of lactation which showed more alveoli and almost twice as many luminal epithelial cells than in control mice (Fig. 2c and d). These results suggest that *RRAS2* overexpression in the mammary gland results in a faster increase in the number of alveolar buds during pregnancy and a slower involution after weaning, therefore showing opposite effects to *Rras2* deficiency when the mammary gland needs to be remodeled: puberty, pregnancy, lactation and involution.

**Fig. 3:**
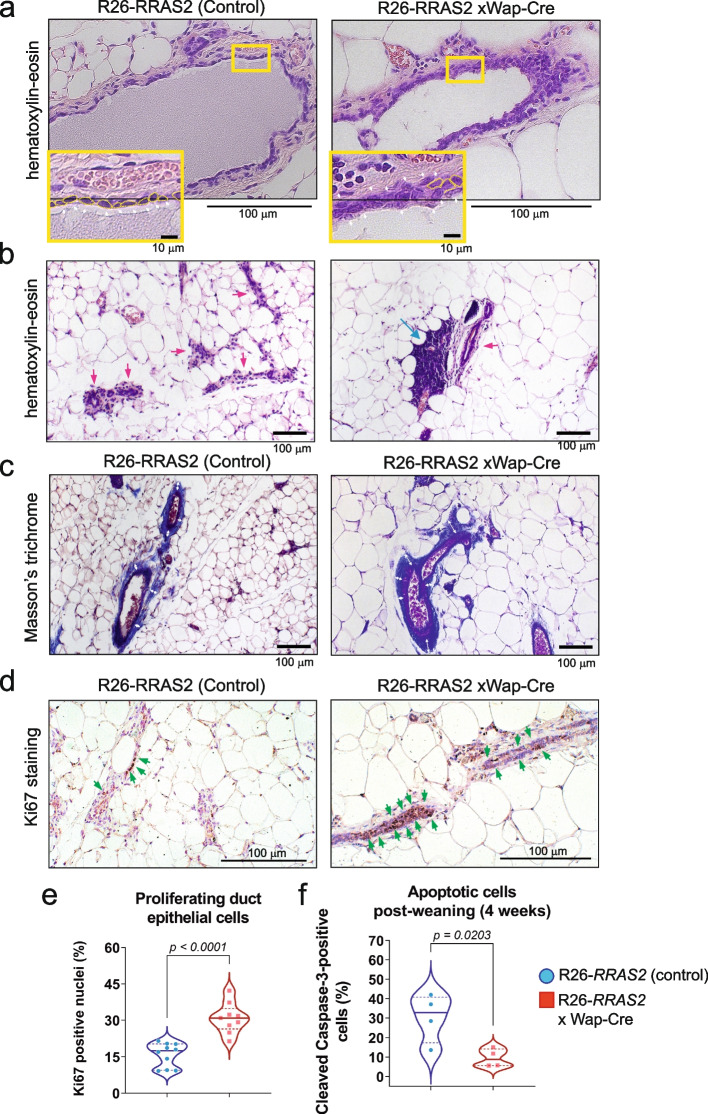
Overexpression of wild-type *RRAS2* causes overproliferation of ductal cells, reduced apoptosis and inflammatory infiltration post-weaning. **a** Hematoxylin and eosin stainings of the right 4th pair mammary gland of control and Rosa26-RRAS2fl/fl x Wap-Cre females in 7-month postpartum females. A representative image per condition is shown at 20 × magnification. Scale bars indicate 100 μm. Insets show magnification of the selected areas framed in yellow to show the presence of more than one layer of epithelial ductal cells (white arrowheads) in *RRAS2*-overexpressing mice. Scale bars indicate 10 μm. **b** Hematoxylin and eosin staining of mammary glands (10 × magnification) as in panel **a** illustrating the presence of mononuclear inflammatory cells (blue arrow) in the adipose tissue, within the vicinity of mammary ducts (red arrows) in *RRAS2*-overexpressing mice. Scale bars indicate 100 μm. **c** Masson’s trichrome staining of mammary glands (10 × magnification) as in panel **a** illustrating the thickened periductal deposition of collagen (white arrows) in *RRAS2*-overexpressing mice. Scale bars indicate 100 μm. **d** Immunoperoxidase and hematoxylin staining of mammary glands (20 × magnification) as in panel **a** showing the presence of Ki67 + proliferating cells (green arrows) in the mammary ducts of control and *RRAS2*-overexpressing mice. **e** Violin plot showing the percentage of Ki67 + nuclei in 9 independent images of 0.2 mm^2^ at 20 × magnification as in panel d. Significance was assessed using two-tailed unpaired t-tests. **f** Violin plot showing the percentage of cleaved caspase-3 + cells in immunoperoxidase and hematoxylin and eosin stainings of mammary glands 4 weeks post-weaning (not shown). Data were generated from four independent images per condition of 0.2 mm^2^ taken at a 20 × magnification. Significance was assessed using two-tailed unpaired t-tests

We next carried out an immunohistochemical study of non-tumoral mammary glands of postpartum Rosa26-*RRAS2*^fl/fl^ x Wap-Cre mice long after the last pregnancy (20 weeks) to determine if *RRAS2* overexpression caused permanent changes in the ductal epithelium that could be considered as premalignant. We found that, unlike in control mice, the ducts of old postpartum Rosa26-*RRAS2*^fl/fl^ x Wap-Cre mice were lined by more than one layer of luminal epithelial cells, in some areas reaching 3–4 layers (Fig. 3a, insets), indicating that *RRAS2* overexpression had caused hyperproliferation of the ductal epithelial cells. In addition, hematoxylin and eosin stainings showed that the breast adipose tissue next to ductal structures was heavily infiltrated by mononuclear cells, suggesting an inflammatory cell infiltration in *RRAS2*-overexpressing mice but not in control mice (Fig. 3b). Using Masson’s trichrome staining, we observed that mammary gland ducts were surrounded by thicker layers of collagen in old postpartum Rosa26-*RRAS2*^fl/fl^ x Wap-Cre mice than in control mice (Fig. 3c). In addition, we stained the mammary gland sections with an anti-Ki67 antibody to assess the number and percentage of proliferating cells within the ductal epithelial cells. We found that a median and mean of 31% of the epithelial cells were proliferating in *RRAS2*-overexpressing mice, twice as many as in control mice (Fig. 3d and e). We finally, stained mammary gland sections of 4-week post-weaning females with an antibody specific for cleaved (active) caspase-3 in order to determine if there were differences in epithelial cell apoptosis during mammary gland involution. We found a significantly reduced frequency of apoptotic cells in *RRAS2*-overexpressing mice (Fig. 3f). Both, the hyperproliferation of epithelial cells and their reduced rate of apoptosis during involution could be responsible for the multilayer cell lining of the mammary gland ducts and, together with the pro-inflammatory environment, eventually cause a pre-neoplastic state of the mammary glands in Rosa26-*RRAS2*^fl/fl^ x Wap-Cre mice. This phenotype results from the overexpression of *RRAS2* from the time of the first pregnancy in Rosa26-*RRAS2*^fl/fl^ x Wap-Cre mice. Next, we interrogated if sexual hormones during pregnancy could be inducing overexpression of the endogenous *Rras2* gene in mice. We administered nulliparous female C57BL/6 mice daily doses of β-estradiol or progesterone to analyze 5 days later if such hormones had induced transcriptional overexpression of the *Rras2* gene. We found no significant effect of either hormone (Fig. S2), suggesting that other hormones regulated *Rras2*. Alternatively, other genes associated to pregnancy and post-partum could be responsible for determining the preneoplastic state once that *RRAS2* is overexpressed by still unknown mechanisms.

### *RRAS2* mRNA is overexpressed in more than two thirds of human breast cancers and associated to Triple Negative ones

To better classify the mouse breast tumors arising in *RRAS2*-overexpressing mice into a molecular subtype and gain a preliminary understanding of the alterations induced by *RRAS2* overexpression, we conducted RNAseq analysis on 15 independent tumors that emerged in 8 Rosa26-*RRAS2*^fl/fl^ mice (2 MMTV-Cre + , 4 Sox2-Cre + , 2 Wap-Cre + : Table 1) and 9 samples of normal mammary gland tissue (2 non-transgenic nulliparous females, 3 non-transgenic breeders, 2 Cre^—^Rosa26-RRAS2^fl/fl^ nulliparous females, and 2 Cre^—^Rosa26-RRAS2^fl/fl^ pregnant females: Table 1).

**Fig. 4:**
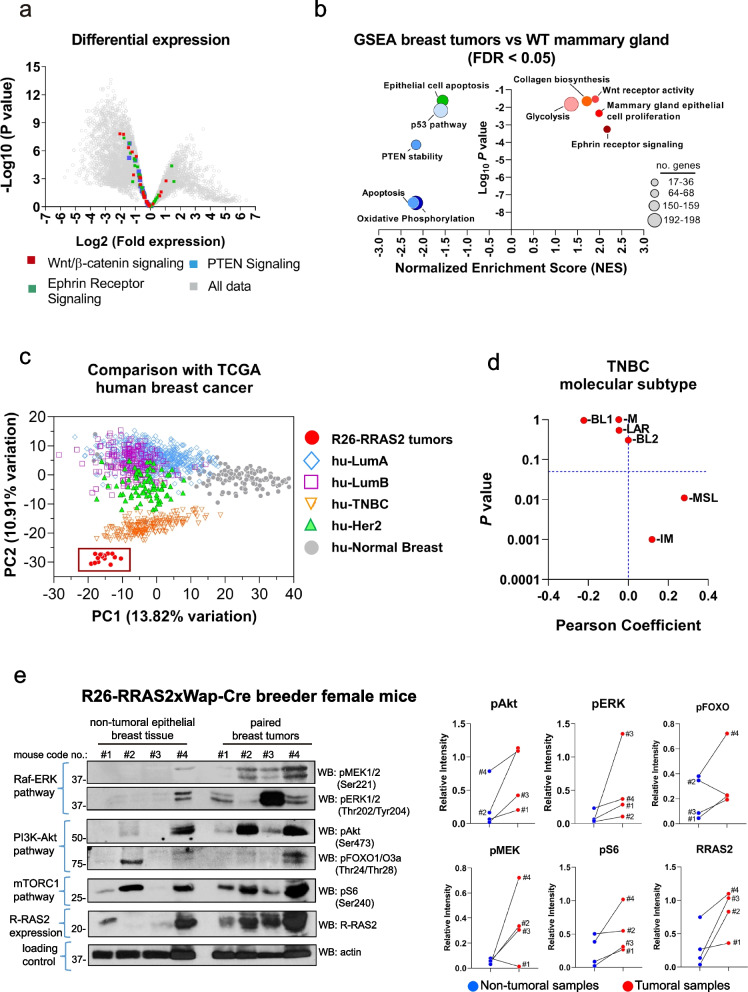
Breast tumors that emerge in *RRAS2*-overexpressing mice are most closely related to human Triple Negative Breast Cancers. **a** Volcano plot showing the *P* value versus the differentially expressed genes in 15 tumor samples as opposed to 9 samples of non-tumor mammary gland tissue. The position of some genes selected according to their participation in the indicated pathways is indicated with colors. **b** Summary results after Gene Set Enrichment Analysis (GSEA) of RRAS2-overexpressing breast tumors compared to normal mammary glands from breeder females. The Normalized Enrichment Score (NES) of the pathway is represented on the x-axis and the adjusted *P* value on the y-axis. Positive NES values mean an enrichment of the pathway in tumor samples, while negative values indicate enrichment in normal mammary glands. Circle size represents the number of genes with significant gene-level statistics in the differential gene expression analysis. FDR: false discovery rate. **c** Principal Component Analysis showing RNAseq data from 15 Rosa26-RRAS2 mouse tumors combined with 1,106 human primary breast tumors and 113 normal mammary tissue samples obtained from the TCGA dataset. The human samples were classified by their molecular subtype. The Rosa26-RRAS2 mouse overexpressing tumors (*n* = 15) are represented as red circles and boxed together. **d** Transcriptomic data from the 15 mouse tumors samples analyzed for subtyping within the TNBC class. The Pearson coefficient is represented on the x-axis and the adjusted *P* value on the y-axis. BL1, Basal-Like 1; BL2, Basal-Like 2; IM, Immunomodulatory; LAR, Luminal Androgen Receptor; M, Mesenchymal; MSL, Mesenchymal Stem-Like. **e** Western blot analysis was performed to assess signaling pathway activity by examining the phosphorylation of key residues in the indicated elements. Post-nuclear cell lysates from freshly isolated tumors of four Rosa26-RRAS2xWap-Cre female breeders, as well as purified epithelial mammary gland cells from non-tumoral glands of the same mice, were analyzed in parallel. The positions of the molecular weight markers are indicated to the left. Line plots to the right show the results of protein band intensity quantification by densitometry. All values were normalized to the actin band intensity. Lines interconnect non-tumoral with tumoral samples from the same mice

Principal Component Analysis (PCA) grouped the 15 tumor samples and clearly differentiated them from the 9 healthy tissue samples (Suppl. Fig. S3a). However, two tumor samples (CCM1 and CCW1: Table 1 and Suppl. Fig.S3a) were considered outliers and excluded from further analysis. A volcano plot illustrates the change in expression in terms of the *P*-value of all the tumor samples relative to all the normal tissue samples (Fig. 4a). Additionally, differences in expression and the differential activation of some genes indicative of signaling pathway activity were studied in tumor and normal tissue through Gene Set Enrichment Analysis (GSEA: Table 2, summarized in Fig. 4b).

Firstly, we observed a significant downregulation of apoptosis (NES score -2.2) which is mirrored by a downregulation of epithelial cell apoptotic processes (NES = -1.5). This is accompanied by the downregulation of pathways mediated by tumor suppressor p53 (NES = -1.5) and PTEN (NES = -2.2). On the other side, tumor formation provoked by *RRAS2* overexpression is accompanied by increased mammary gland epithelial cell proliferation (NES = + 2.1) and by an increase in extracellular matrix (collagen) biosynthesis and modifying enzymes (NES = + 1.8). In terms of activation of pro-oncogenic pathways important for breast cancer, GSEA analysis detected a clear increase in the activity of the Ephrin Receptor (NES = + 2.3) and the Wnt Receptor (NES = + 2.0). Interestingly, we found a hallmark of downregulation of oxidative phosphorylation (NES = -2.1) and an upregulation of glycolysis (NES = + 1.5), which could be related to the known Warburg effect of increased aerobic glycolysis in tumor cells, including breast cancer [38]. In summary, the results of GSEA analysis of transcriptomics (Fig. 4b) largely correspond with the immunohistochemical findings in mammary gland remodeling during pregnancy and post-partum (Figs. 2 and 3) indicating enhanced proliferation, decreased apoptosis and remodeling of the extracellular matrix.

The RNAseq data was also compared with transcriptomic data deposited in the TCGA human breast cancer database to determine which molecular human breast cancer subset is more similar to the mouse breast cancer *RRAS2*-overexpression model. PCA analysis classified human breast cancers into four clusters, all clearly distinguishable from human healthy breast epithelium (Fig. 4c). The Luminal A and Luminal B tumors were the most closely related, followed by the HER2 + tumors, and finally, the Basal or Triple Negative tumors, which were the most clearly distinguished. The 15 mouse tumor samples grouped closer to the Triple Negative breast cancers than to any of the others (Fig. 4c), thus confirming our immunohistochemical data (Fig. 1c, Fig. S1b), showing that the breast tumors emerging in mice overexpressing *RRAS2* most closely resemble the TNBC subtype. This finding in mice overexpressing *RRAS2* is paralleled by the analysis of *RRAS2* expression in human breast cancer from the TCGA database. Accordingly, human tumors with highest expression of *RRAS2* are those with less ER (*ESR1*), less *PGR*, and less *ERBB2* (Suppl. Fig. S4). In addition to clustering by PCA analysis, we compared the 15 independent murine breast tumors emerging in *RRAS2*-overexpressing mice with the TCGA human breast cancer dataset by unsupervised clustering using the R package DESeq2 [26] as described in the Methods section. A heatmap plot is shown in Suppl. Fig. S5. This hierarchical clustering shows again that the murine tumors resemble the basal (TNBC) human breast cancer more than any other molecular subtype.

The transcriptomic data from the 15 mouse tumors samples was subsequently analyzed using the tool developed by Chen et al. (https://cbc.app.vumc.org/tnbc/prediction.php) [30] for subtyping within the TNBC class. The result showed that the TNBC tumor type developed in RRAS2-overexpressing mice resemble more the mesenchymal stem-like (MSL) subtype than any of the other (Fig. 4d). MSL subtype TNBC is characterized by elevated expression of genes related to epithelial-mesenchymal transition (EMT) [29].

In our previous study, we investigated the effects of expressing the R-RAS2 protein with the activating mutation G23V in human and murine breast cancer cell lines on RAS signaling pathways. We discovered that R-RAS2(G23V) expression activated the PI3K/Akt pathway without affecting the Raf-ERK MAPK pathway [13]. In this study, we explored the activation of these pathways in freshly isolated breast tumors resulting from the overexpression of wild-type *RRAS2*. We analyzed the phosphorylation levels of key components of the PI3K/Akt, Raf-ERK, and mTORC1 pathways using western blotting in breast tumors from four Rosa26-RRAS2fl/fl x Wap-Cre breeder female mice and compared them with paired non-tumoral mammary glands from the same mice. Our findings revealed activation of the Raf-ERK, PI3K/Akt, and mTORC1 pathways in all four tumors, although there was considerable variability compared to the paired non-tumoral breast tissue (Fig. 4e). R-RAS2 protein was overexpressed in all four tumors compared to their paired non-tumoral tissues. Notably, non-tumoral epithelial breast tissue from mouse #1, and especially from mouse #4, showed high R-RAS2 expression levels compared to mice #2 and #3. This overexpression might explain the high levels of pAkt(Ser473) observed in the non-tumoral tissue of mouse #4. Another striking observation was that the four tumors had high levels of phosphorylation of MEK1/2 and ERK1/2 in the Raf-ERK pathway and high levels of phosphorylation of Akt, but the tumors with highest phosphorylation in one of the pathways was not coincident with the tumors with the highest phosphorylation in the other. Specifically, tumors #2 and #4 showed the highest pAkt levels, while tumor #3 had the highest pERK1/2 levels, and tumor #4 had the highest pMEK1/2 levels (Fig. 4e). In summary, our results indicate that R-RAS2 overexpression is associated with increased activity of both the Raf-ERK and PI3K/Akt pathways as well as the mTORC1 pathway in freshly isolated breast tumors. Additionally, the downstream pathway activation resulting from R-RAS2 overexpression does not follow a single uniform pattern.

**Fig. 5:**
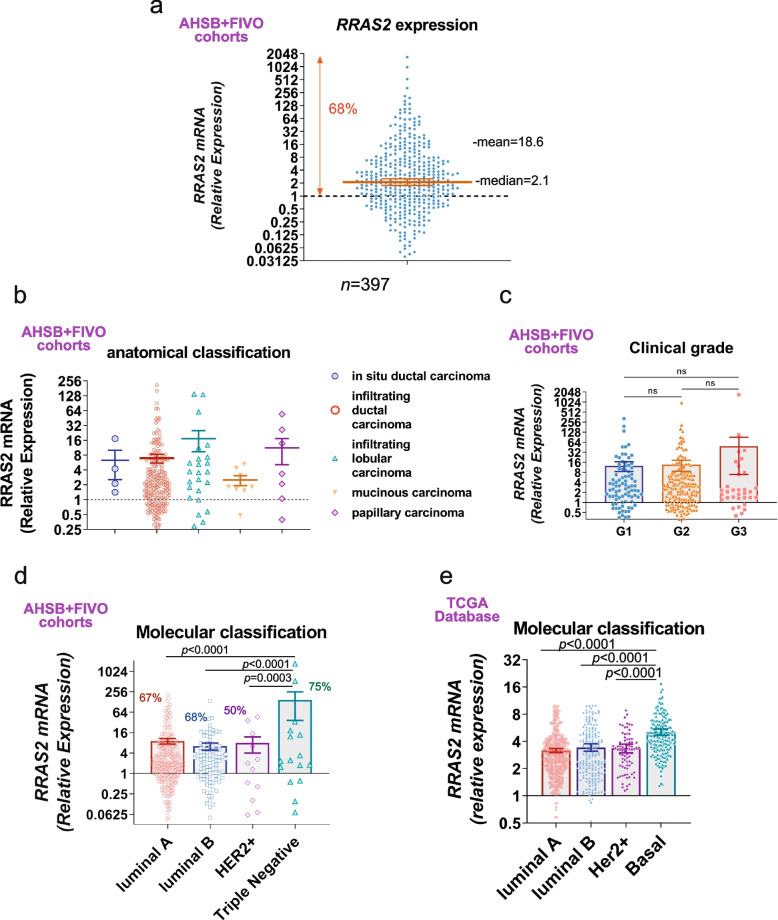
*RRAS2* is frequently overexpressed in all types of human breast cancer but more especially in TNBC. **a** Column plot showing all points (*n* = 397) of the aggregated AHSB + FIVO cohorts with the relative *RRAS2* mRNA expression in log2 scale referred to the mean of *n* = 10 non-tumoral human breast tissue set as 1. The median ± 95% CI is shown as a line and error. A total of 68% of the samples had a relative *RRAS2* mRNA expression > 1. **b** Column plot showing the mean ± s.e.m. and all points of the aggregated AHSB + FIVO cohorts classified according to their pathological type. **c** Column plot showing the mean ± s.e.m. and all points of the aggregated AHSB + FIVO cohorts classified according to their clinical grade. **d** Column plot showing the mean ± s.e.m. and all points of the aggregated AHSB + FIVO cohorts divided according to their molecular classification. Significance was assessed using an ordinary One-way ANOVA Tukey’s multiple comparison test. **e** Column plot showing the mean ± s.e.m. and all points (1,160) of the normalized *RRAS2* expression in breast cancer samples of the TCGA dataset classified by their molecular type. Significance was assessed using an ordinary One-way ANOVA Tukey’s multiple comparison test

The forced overexpression of *RRAS2* in ductal epithelial cells leads to the development of breast cancer in a causal relationship (Fig. 1). To study if *RRAS2* overexpression could also be a driver oncogene in the development of human breast cancer, we collected a total of *n* = 397 human breast cancer samples from two biobanks in Spain (AHSB and FIVO) and analyzed *RRAS2* overexpression (Table 3). Total RNA was extracted from frozen tissue samples, and *RRAS2* mRNA expression was measured by RT-qPCR, with the values referred to the mean of expression in 10 normal healthy breast samples (set as 1, Fig. 5a). The median and 95% CI were 2.1 (1.8–2.5), and the mean ± SD was 18.6 ± 106. Sixty-eight percent of the breast tumors overexpressed *RRAS2*, with tumors expressing hundreds or even one thousand-fold the levels found in normal tissue (Fig. 5a). The grouping of breast tumor samples in the AHSB + FIVO cohorts, according to their pathological classification, showed that *RRAS2* overexpression was found in all types of tumors, from in situ to infiltrating carcinomas (Fig. 5b). Overexpression of *RRAS2* was also found to be independent of clinical grade (Fig. 5c). According to their molecular classification, RRAS2 was found overexpressed in all types of tumors in the AHSB + FIVO cohorts, but more frequently in TNBC (75%) and have the highest mean of expression (Fig. 5d). In agreement with our findings, Basal-type breast cancers are the ones with the highest expression of *RRAS2* in the TCGA metadata (Fig. 5e). In summary, these results show that more than two-thirds of human breast cancers overexpress *RRAS2*, and therefore, overexpression of this gene could be behind the development of breast cancer. Additionally, the human data shows that overexpression is highest within the Triple Negative type of breast cancers, in correlation with the type of tumor generated in the mouse Rosa26-*RRAS2* model.

### Higher RRAS2 mRNA expression in breast cancer is associated with a worse prognosis

**Fig. 6:**
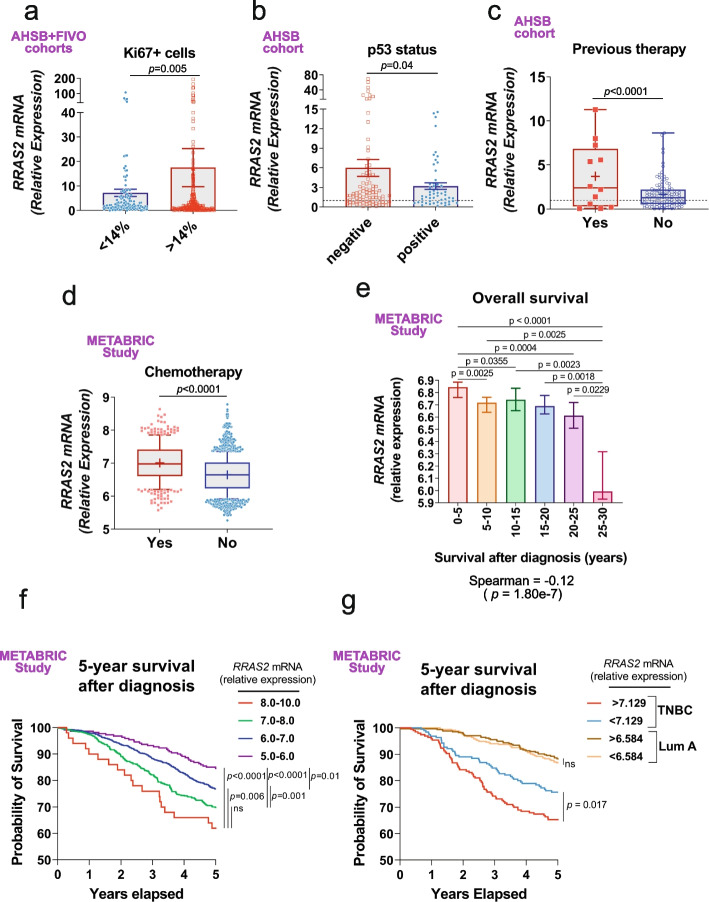
Higher *RRAS2* expression in human breast cancer is associated to worse prognosis. **a** Column plot showing all datapoints and the mean ± s.e.m. for *RRAS2* relative mRNA expression in all samples of the aggregated AHSB + FIVO cohorts classified according to having a percentage of Ki67 + > 14% or lower. Outliers were excluded based on a ROUT (Q = 0.1) method. Significance was assessed using an unpaired two-tailed t-test with Welch’s correction. **b** Column plot showing the all datapoints and the mean ± s.e.m. for *RRAS2* relative mRNA expression in all samples of the AHSB cohort classified according to being positive or negative for p53 determined by immunohistochemistry. Outliers were excluded based on a ROUT (Q = 0.1) method. Significance was assessed using an unpaired two-tailed t-test with Welch’s correction. **c** Box and whisker plots showing all the points, the median and mean ( +) values for relative *RRAS2* mRNA expression in tumor samples from the AHSB cohort classified according to having received previous anti-cancer therapy or not. Significance was assessed with a two-sided unpaired t-test with Welch’s correction after excluding outliers using the ROUT method (Q = 0.1%). **d** Box and whisker plots showing the 10–90 percentile, the median and the mean ( +) values of normalized *RRAS2* mRNA expression microarray data in breast cancer samples (METABRIC study; 1,904 samples) classified according to having received anti-cancer chemotherapy or not. Significance was assessed with a two-sided unpaired t-test with Welch’s correction. **e** Column plot showing the median ± 95% CI of normalized *RRAS2* mRNA expression in BC samples (METABRIC study; 1,904 samples) classified according to overall survival after diagnosis (in years) grouped as indicated. Significance was assessed with a one-way ANOVA Tukey’s multiple comparison test. A negative Spearman’s rank correlation coefficient of -0.12 reflects an inverse relationship between *RRAS2* expression and survival with a *p* = 1.80e.^−07^ **f** Percentage survival plot showing 5-year survival probability of patients from the METABRIC study in panel e classified according to four intervals of *RRAS2* mRNA expression. Significance was assessed using a Log-rank (Mantel-Cox) test. ns, not significant *p* > 0.05. **g** Percentage survival plot showing 5-year survival probability of patients from the METABRIC study in panel e with tumors diagnosed as TNBC or Luminal A. Tumor samples under each category were further classified as high *RRAS2* RNA expressors (above the median; > 7.129 for TNBC, > 6.584 for Luminal A) and low expressors (below the median). Significance was assessed using a Log-rank (Mantel-Cox) test. ns, not significant *p* > 0.05

Ki67, a proliferation marker commonly assessed by immunohistochemistry in breast cancer, holds significant value in subtype classification, prognosis, and response to therapies, with 14% of Ki67 + cells commonly accepted as a cutoff point [39]. The immunohistochemical classification of breast tumor samples within the combined AHSB + FIVO cohort into Ki67 + low (< 14%) and Ki67 + high (> 14%) groups showed that *RRAS2* expression was significantly higher in the Ki67 + > 14% group, suggesting that higher *RRAS2* expression in breast cancer tumors is associated with a worse prognosis (Fig. 6a).

Another marker of prognosis is the expression or absence of the tumor suppressor protein p53. The absence of protein by immunohistochemistry is commonly associated with a *TP53* gene mutation and, therefore, loss of function [40]. In the AHSB cohort, we observed a significantly stronger overexpression of *RRAS2* in p53-negative compared to p53-positive breast cancer samples (Fig. 6b). Additionally, *RRAS2* showed a significantly higher expression in tumor samples obtained from patients relapsing after therapy compared to those obtained before therapy (Fig. 6c). Similarly, higher *RRAS2* expression was found in the METABRIC data associated with samples from patients who received chemotherapy compared to the sample set of untreated patients (Fig. 6d). Finally, although we lacked survival data in the AHSB or FIVO cohorts, analysis of the METABRIC data revealed a strong correlation between higher *RRAS2* expression in breast tumors and shorter overall survival after diagnosis (Fig. 6e). This was particularly evident for the 5-year survival rate after diagnosis (Fig. 6f). The lower survival rate for patients with tumors expressing the highest levels of *RRAS2* mRNA could be attributed to the fact that TNBC exhibits a poorer prognosis and higher *RRAS2* expression (Fig. 5d). While this is a clear factor contributing to poorer survival, it is not the sole determinant. Indeed, when considering only the data from the METABRIC dataset corresponding to TNBC, a significantly shorter survival rate was observed for patients with TNBC expressing *RRAS2* above the median compared to those below the median (Fig. 6g). Such differential survival rate was not observed in the group of patients diagnosed with luminal A type breast cancer. All these data indicate that *RRAS2* overexpression is a marker of worse prognosis in human breast cancer.

### A single nucleotide polymorphism (SNP) in the 3’-UTR region of *RRAS2* and locus amplification strongly associate this gene with the development of human breast cancer

The rs8570 polymorphism in the 3’UTR of the *RRAS2* mRNA involves the replacement of a G residue by a C at position + 124, downstream of the STOP codon. We previously found a frequency of CC homozygotes at the SNP position slightly above the expected for a Hardy–Weinberg equilibrium in CLL patients, at the expense of the frequency of GC heterozygotes but not of GG homozygotes (18). Such disequilibrium in favor of the C allele was not found in a cohort of healthy human blood donors [41].

**Fig. 7:**
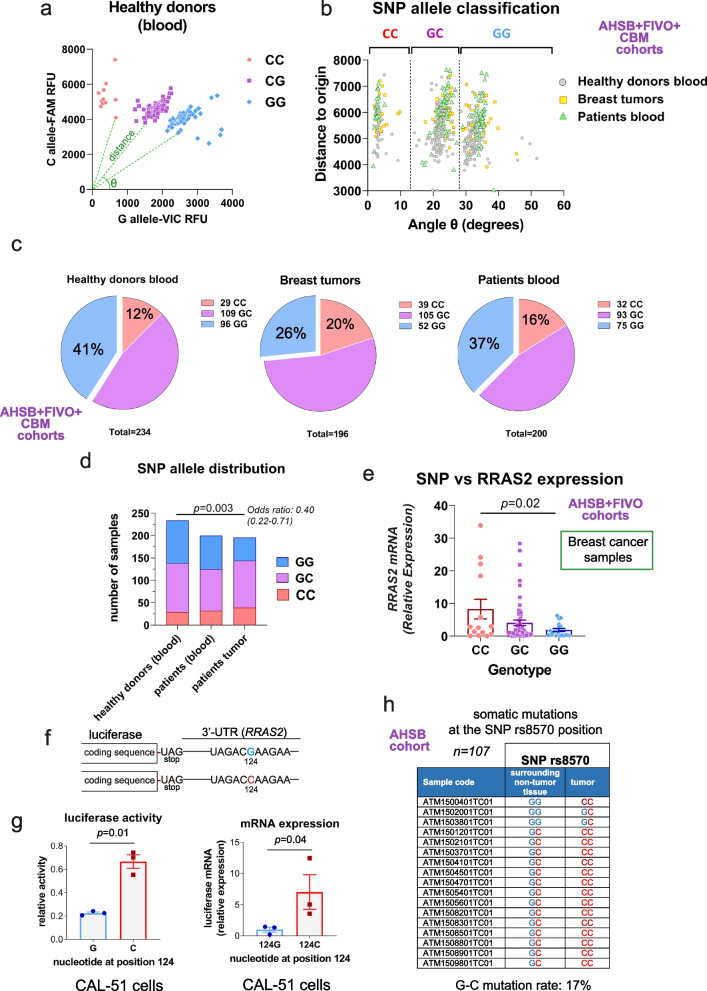
The C allele at the rs8570 SNP in the 3’UTR of *RRAS2* strongly associates this gene with breast cancer. **a** XY plot illustrating how the angle θ and the distance to the origin is calculated for every sample of genomic DNA after qPCR using two Taqman (FAM and VIC) probes. The example shows some results corresponding to blood from healthy donors. **b** XY plot showing the angle θ versus the distance to origin, calculated as in panel a, for every breast tumor, healthy donor blood sample and blood samples from breast cancer patients. Tumor samples were from the AHSB and FIVO cohorts, BC patient blood samples from the FIVO cohort and healthy blood samples from the CBM and IMIM cohorts. **c** Pie chart representation of the distribution of the GG, GC and CC genotypes at the rs8570 SNP in all samples analyzed as in panel b. The number of samples is indicated for each one of the classifications, and the percentage of the total for the CC and GG genotypes. The total number of samples is indicated under each chart. **d** Stacked bar plot showing the distribution of GG, GC and CC genotypes in the three types of blood and tumor samples. A contingency Fisher’s exact test shows the significance of the differences in the distribution of GG vs CC homozygotes in tumors compared to healthy blood samples. **e** Scatter plot showing all points and the mean ± s.e.m. of *RRAS2* mRNA expression in tumor samples from the AHSB plus FIVO cohorts classified according to the SNP genotype. Significance was assessed with a one-way ANOVA Tukey’s multiple comparison test after excluding outliers using the ROUT method (Q = 0.5%). **f** Schematic representation of the two constructs containing the 3’UTR of *RRAS2* appended to the luciferase reporter construct. The only difference between the two constructs was the presence of a G or a C at the SNP rs8570 position. **g** Bar plot showing all data points and the mean ± s.e.m. of luciferase activity (left) and mRNA expression (right) in lysates of human CAL-51 BC cells transfected with the two reporter constructs shown in panel f. Significance was assessed using a two-sided unpaired t-test and Welch’s correction. **h** Enumeration of the somatic mutations detected at the SNP rs5870 position in the tumor samples (*n* = 107) from the AHSB cohort relative to the surrounding matched non-tumor mammary tissue

To determine if the frequency of the rs8570 SNP was altered in breast cancer, we analyzed the expression of the G and C alleles of this SNP by a dedicated PCR approach [42] using tumor samples from our AHSB and FIVO cohorts, blood samples from breast cancer patients in the FIVO cohort, and blood samples from healthy human volunteers in two cohorts (Table 3). To classify the samples among GG and CC homozygotes or GC heterozygotes, we plotted the qPCR fluorescence signals corresponding to the C and G alleles (an example is in Fig. 7a) and calculated the angle θ to the x-axis and the distance to the origin for each datapoint. The angle θ allowed to classify all samples into three groups corresponding to CC, GC, and GG genotypes (Fig. 7b). The distance to the origin was generally longer for breast tumor samples and blood samples from BC patients than for blood of healthy donors (Fig. 7b). Such increased distance to the origin points out to a higher than normal abundance of genomic DNA encoding the *RRAS2* gene in both tumor and blood from BC patients as discussed below.

According to the genotype classification into CC, GC, and GG groups, there was a tendency to have more CC homozygotes and fewer GG homozygotes in breast cancer samples than in blood from healthy donors (Fig. 7c), with blood samples from BC patients showing an intermediate distribution. Using a contingency test, we could determine that the frequency of CC and GG homozygotes was significantly different between the breast tumor samples and the healthy blood donor samples (Fig. 7d). As for CLL [19], we could correlate expression of the C allele in homozygosity with a higher expression of the *RRAS2* mRNA in the breast tumor samples (Fig. 7e).

To determine whether the C allele favors the overabundance of *RRAS2* mRNA in BC tumor samples, a luciferase expression construct was generated in which the 3’UTR region of human *RRAS2* mRNA with either a G or C residue at the SNP position was fused downstream of the cDNA encoding the reporter gene luciferase (Fig. 7f). When expressed in the Triple Negative breast cancer cell line CAL-51, we found stronger luciferase activity in the case of the construct bearing the C allele than in that bearing the G one (Fig. 7g). Such higher luciferase activity for the construct bearing the C allele was paralleled by a higher mRNA content (Fig. 7g). The two luciferase constructs were additionally tested in two other human breast cancer cell lines (BT549 and MCF7) and in three human leukemic cells lines (Jurkat, HUT-78 and MEC-1) and, in all cases, the construct bearing the C allele was expressed at higher levels than the G-containing one (Suppl. Fig. S6). These results indicated that expression of the C allele alone can enhance the abundance of *RRAS2* mRNA and explain the effect of the allele on human breast cancer.

Another finding linking the C allele at the SNP rs5870 position and breast cancer came from the analysis by Sanger sequencing of 107 paired samples of breast tumors and non-tumoral surrounding breast tissue from the same patients in the AHSB cohort. We found a disparity in the sequences in 17% of the positions, showing the presence of G > C somatic mutations at the SNP position (Fig. 7h). Taken together, the data showing SNP C allele enrichment, higher *RRAS2* expression for the C allele, and the existence of G > C mutations all point out to *RRAS2* as a relevant gene driving breast cancer in humans.

**Fig. 8:**
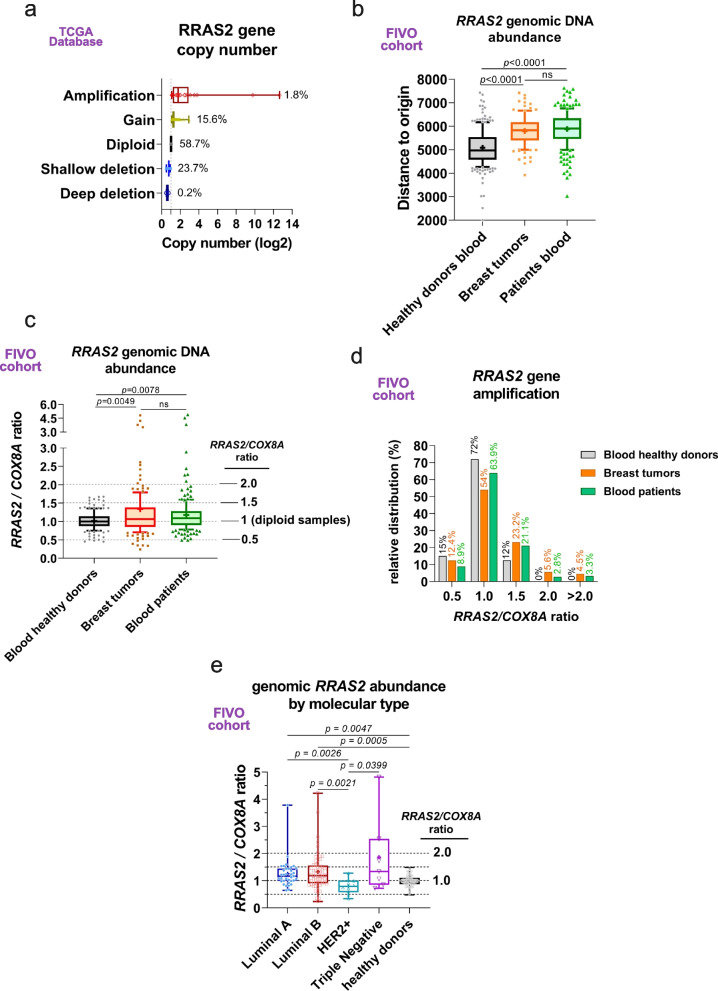
The *RRAS2* gene is frequently amplified in tumoral and non-tumoral tissues from breast cancer patients. **a** Box and whiskers plot of breast cancer samples from the TCGA database classified according to the *RRAS2* gene copy number. **b** Box and whiskers plot showing the median, the mean ( +), and the 10–90 percentile of the distance to the origin calculated from Taqman® PCR results of Fig. 7A and B. Significance was assessed with a one-way ANOVA Tukey’s multiple comparison test. **c** Box and whiskers plot showing the median, the mean ( +), and the 10–90 percentile of the ratio between *RRAS2* gene and *COX8A* gene abundance measured by qPCR of genomic DNA. Values corresponding to diploid samples (2 copies of *RRAS2* and 2 copies of *COX8A* genes) are assumed to be the median and the mean of the ratios found in the healthy volunteer blood samples. Significance was assessed with a one-way ANOVA Tukey’s multiple comparison test. **d** Column plot showing the distribution of all genomic DNA samples according to the three categories (blood from healthy donors, blood from breast cancer patients and breast cancer samples) and the RRAS2/COX8A ratio. **e** Box and whiskers plot showing the median, the mean ( +), and all data points of the *RRAS2*/*COX8A* ratios for the FIVO (tumor samples) and CBM (healthy) genomic DNA samples divided according to their molecular classification. Significance was assessed using a One-way ANOVA Kruskal–Wallis test

Unlike somatic mutations in the 3’UTR of the *RRAS2* gene described just above, no mutations have been described at a significant rate in the coding sequence of *RRAS2* in breast cancer. By contrast, copy number alterations of the *RRAS2* locus are reported, resulting in nearly 18% gains and amplifications (Fig. 8a). Such an increase in copy number could be related to overexpression of the wild type *RRAS2* gene in breast cancer. In this regard, we reasoned that the distance to the origin in the G vs. C SNP plots of Fig. 7a and b could be a reflection of the abundance of the *RRAS2* gene in genomic DNA. By grouping all the distance data for the three types of samples: blood from healthy donors, breast tumors, and blood from BC patients, we found a significant increase for both the tumor and blood samples from BC patients compared to the blood samples from healthy volunteers (Fig. 8b). This data suggests that the *RRAS2* gene is not only amplified in breast tumors but also in the blood of patients. Here, we need to point out that we have analyzed genomic DNA from total blood and therefore the results of *RRAS2* gene abundance derive from total non-transformed blood cells and not from a minority of circulating tumor cells. Consequently, the results of gene abundance in blood from BC patients suggest that *RRAS2* amplification is prior to BC development. To determine the presence of amplified *RRAS2* gene in tumor and normal tissue (blood) samples from BC patients we also used an alternate approach: we measured the abundance of genomic *RRAS2* using qPCR primers that anneal to the coding sequence of *RRAS2* in a specific manner and referred it to the abundance of genomic *COX8A*. *RRAS2* is encoded in the short arm of chromosome 11 (11p5.2) and *COX8A* in the long arm (11q13.1), near the centromere. *COX8A* is a house-keeping gene that encodes for a cytochrome c oxidase component with low cancer specificity (https://www.proteinatlas.org/ENSG00000176340-COX8A). By referring *RRAS2* to *COX8A* we could determine the level of amplification of *RRAS2*, knowing that we would miss those samples in which the entire chromosome 11 is amplified. The median and mean for the *RRAS2/COX8A* ratio in genomic DNA of healthy blood donors were 1, and therefore, we assume those correspond to diploid samples, i.e., 2 copies of *RRAS2* gene per cell (Fig. 8c). The median for tumor and blood samples from BC patients was 1.06 and 1.08, respectively, and the mean and 95% CI was 1.34 (1.04–1.63) for tumor samples and 1.15 (1.08–1.22) for patients’ blood samples. Overall, the results of Fig. 8c showed a significant amplification of the *RRAS2* gene in both tumor and blood samples from BC patients when referred to the blood of healthy volunteers. A calculation of the percentages of samples with amplification showed that 23.2% of tumors and 21.1% of patients’ blood samples had 1.5-fold higher number of copies of *RRAS2* gene than the *COX8A* gene, against 12% in blood from healthy volunteers (Fig. 8d). The difference in terms of percentage was even clearer when we considered the percentage of samples with an *RRAS2/COX8A* gene ratio of 2.0 and higher: a total of 10.1% of the tumors and 7.8% of the patients’ blood samples compared to 0% for the healthy donors’ blood. These data confirm those of Fig. 8b suggesting that a sizeable percentage of not only breast tumors but also of non-tumoral tissue (blood) in BC patients have amplified the *RRAS2* gene and are suggestive of a pre-existing breast cancer-prone condition. In regard to their molecular classification, the breast tumor samples with significant *RRAS2* gene amplification were Luminal A and Luminal B, but not HER2 + ones (Fig. 8e). Triple Negative BC tumors had significant amplification of *RRAS2* when compared to the HER2 + ones, but the number of samples was too low as to achieve significance when compared to samples from healthy donors.

### Highest RRAS2 overexpression in breast cancer is associated with parity

**Fig. 9:**
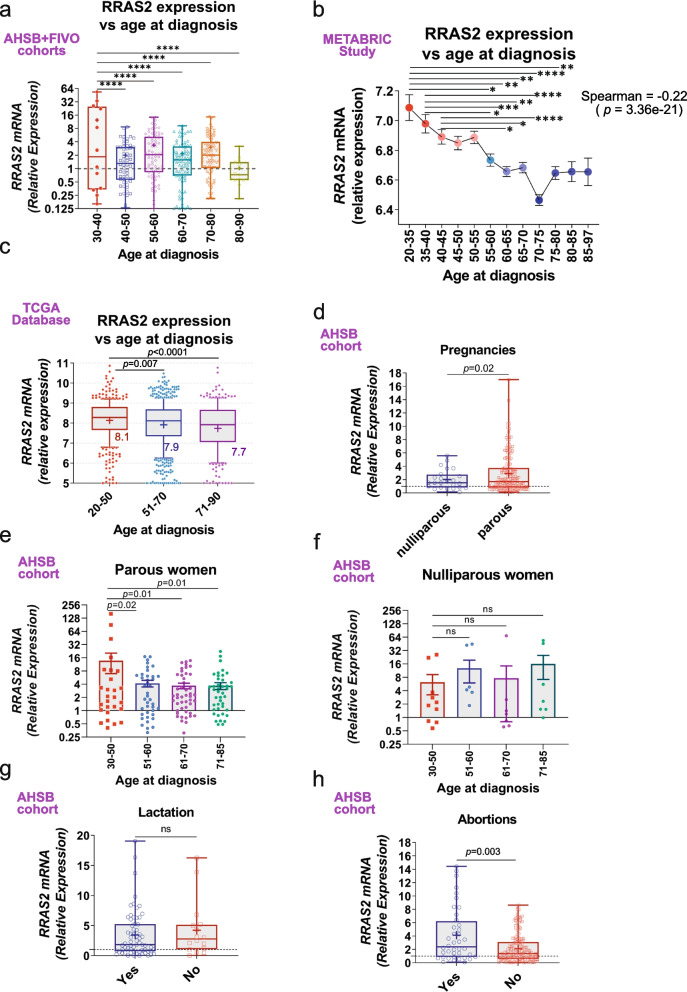
Highest *RRAS2* expression is found in tumors from young parous breast cancer patients. **a** Box and whiskers plot showing the median, the mean ( +), and all data points of *RRAS2* mRNA expression in tumors from the aggregated AHSB and FIVO cohorts classified according to the age of the patients at diagnosis. Significance was assessed using a One-way ANOVA Tukey’s multiple comparisons test after excluding outliers using the ROUT method (Q = 0.1%). **** *p* < 0.0001. **b** Mean ± s.e.m. of normalized *RRAS2* expression in breast cancer tumors from the METABRIC study (*n* = 1,904) classified according to the age at diagnosis. Significance was assessed with a one-way ANOVA Kruskal Wallis' multiple comparisons test. * *p* < 0.05; ** *p* < 0.01; *** *p* < 0.001; **** *p* < 0.0001. A negative Spearman’s rank correlation coefficient of -0.22 reflects an inverse relationship between *RRAS2* expression and age at diagnosis with a *p* = 3.36e^−21^ **c** Box and whiskers plot showing the median, the mean ( +), and the 10–90 percentile of RRAS2 mRNA expression in breast cancer samples from the TCGA database classified according to the age of the patients at diagnosis. Significance was assessed with a one-way ANOVA Kruskal–Wallis' multiple comparisons test. **d** Box and whiskers plot showing all points, the median and the mean ( +) *RRAS2* relative expression values for tumors samples of the AHSB cohort obtained from parous and nulliparous patients. Significance was assessed using a two-tailed unpaired t-test with Welch’s correction. **e** Column plot showing all points and the mean ± s.e.m. of *RRAS2* mRNA expression in parous patients from the AHSB cohort classified according to the age at diagnosis. Significance was assessed using a one-way ANOVA Dunnett’s multiple comparison test. **f** Column plot showing all points and the mean ± s.e.m. of *RRAS2* mRNA expression in nulliparous patients from the AHSB cohort classified according to the age at diagnosis. Significance was assessed using a one-way ANOVA Dunnett’s multiple comparison test. **g** Box and whiskers plot showing all points, the median and the mean ( +) *RRAS2* relative expression values for tumors samples of the AHSB cohort classified according to patients having breastfed or not. Significance was assessed using a two-tailed unpaired t-test with Welch’s correction. **h** Box and whiskers plot showing all points, the median and the mean ( +) *RRAS2* relative expression values for tumors samples of the AHSB cohort classified according to the existence of miscarriages or not. Significance was assessed using a two-tailed unpaired t-test with Welch’s correction

The AHSB cohort contains data on the number of pregnancies, age of diagnosis, and lactation, which could be relevant when considering possible associations between *RRAS2* overexpression and breast cancer (BC) (Table 3). We first observed an association between BC diagnosed in women younger than 40 years old and the strongest *RRAS2* expression (Fig. 9a). This relationship between early age at diagnosis and higher *RRAS2* expression was also found after analysis of data in the METABRIC study: a clear inverse relationship was found, with a peak of expression in youngest women (20–35 years at diagnosis, Fig. 9b). An inverse association between age at diagnosis and *RRAS2* expression was also found in the metadata analysis of the TCGA study (Fig. 9c). The higher *RRAS2* expression found in the groups of younger women could be a consequence of the higher frequency of TNBC in young patients. Indeed, if tumor samples in the METABRIC study are classified according to their molecular type, more than 50% of tumors diagnosed in women between the ages of 30 and 40 corresponded to the TNBC type (Suppl. Fig. S7a and S7b). However, molecular classification was not the only reason to explain the highest *RRAS2* mRNA expression in younger patients because, regardless of their molecular classification, all tumors showed an inverse relationship between *RRAS2* expression and the age of the patient (Suppl. Fig. S7c). We suspected that stronger *RRAS2* expression in tumors diagnosed in younger women might also be related to pregnancy and/or lactation. Indeed, we found significantly stronger expression of *RRAS2* mRNA in tumors isolated from parous patients than from nulliparous patients in the AHSB cohort (Fig. 9d). Among parous breast cancer patients, those with the highest expression of *RRAS2* in their tumors were those diagnosed at an age of 50 or younger (Fig. 9e). Such an effect was not seen in the group of nulliparous patients, in which *RRAS2* expression does not seem to be age-related (Fig. 9f). These data further point to an association between tumors with the highest *RRAS2* expression and pregnancy. *RRAS2* expression does not seem to be linked to breastfeeding (Fig. 9g) but is significantly higher in patients who had miscarriages than in those who didn’t (Fig. 9h), suggesting that rather than with lactation, the highest *RRAS2* expression in BC is related to pregnancy. Altogether, these data suggest that, although *RRAS2* overexpression is common even in BC diagnosed in nulliparous women, it is higher in tumors that could be related to childbearing.

## Discussion

We demonstrate here that the overexpression of wild type *RRAS2* is sufficient to drive the development of breast ductal adenocarcinomas in 100% of female mice undergoing cycles of gestation and lactation. By contrast, less than 5% of nulliparous females of the three *RRAS2*-overexpressing mouse lines developed breast tumors. These data establish *RRAS2* as a driver oncogene that, unlike the classic *RAS* genes, does not require activating mutations to trigger the development of a major cancer. We also demonstrated a driver role for *RRAS2* in malignant transformation in chronic lymphocytic leukemia (CLL) [19].

The question arising from the mouse data is whether the overexpression of wild-type *RRAS2* contributes to the development of human breast cancer. Initial data indicated that 7 out of 9 human breast cancer cell lines expressed high levels of R-RAS2 protein [11]. In our analysis of mRNA expression in *n* = 397 breast cancer samples, we found that 68% overexpress the *RRAS2* gene compared to the mean of non-tumoral breast tissue samples, with a mean of 18.6 and a median of 2.1. Could this level of overexpression be sufficient to cause mammary epithelial cell transformation? The analysis of *RRAS2* expression in non-tumoral mammary gland tissue of Rosa26-RRAS2fl/fl x Sox2-Cre mice shows a mean 3.2-fold above the expression of the murine *Rras2* gene in non-tumor developing control breeders. This indicates that *RRAS2* can initiate the development of breast cancer if moderately overexpressed. Another argument is that the *Rras2* gene is haploinsufficient, as heterozygote *Rras2*^+/—^ mice have the same phenotype as fully knockout *Rras2*^—/—^mice, suggesting tight control of the *Rras2* gene dose [16]. Accordingly, overexpression of *RRAS2* could underlie the development of more than two-thirds of human breast cancers. This is currently a correlation; however, two sets of genetic data strongly support the evidence of a causal relationship: the frequency of the C allele at the SNP rs8570 position in the 3’UTR of *RRAS2* in breast tumors, and the overexpression of the *RRAS2* gene in a non-tumoral tissue (blood) of breast cancer patients.

We demonstrate here that CC homozygosity at the SNP position is found at a significantly higher proportion than GG homozygosity in breast tumor samples compared to that in blood from the healthy population. The dose of the C allele correlates with higher mRNA expression, a relationship also found in CLL. Additionally, we identified a 17% G > C mutation rate at the SNP position in tumor samples compared with surrounding non-tumoral mammary tissue in a group of *n* = 107 paired samples. This also suggests *RRAS2* overexpression as a driver of human breast cancer. The second important piece of genetic evidence supporting a cause-effect relationship between *RRAS2* overexpression and breast cancer is that we found the gene to be amplified in the blood of approximately 28% of breast cancer patients. This suggests that amplification of the gene in this subset of patients precedes breast cancer development. Since we show that higher *RRAS2* overexpression is linked to worse prognosis and shorter survival, the analysis of the SNP allele composition and of gene amplification could become useful prognostic tools in patient staging and classification. Although *RRAS2* is overexpressed in the majority of breast cancers, independent of their pathological classification, clinical grade, and molecular classification, the overexpression is highest and more frequent in TNBC, the type of BC for which there are fewer effective therapeutic options nowadays due to its unresponsiveness to anti-hormone therapies and anti-HER2 antibodies. In our cohorts, 75% of the TNBC samples overexpress *RRAS2*. Interestingly, the breast tumors that develop in our two *RRAS2*-overexpressing mouse lines correspond, according to immunohistochemical markers and the global transcriptome, to the basal subset of TNBC in humans, thus confirming the causal effect of *RRAS2* overexpression in TNBC.

Notably, childbearing is transiently associated with a higher incidence of BC compared to nulliparous women of the same age. In addition, TNBC is more prevalent in younger women, who have a twofold higher risk of developing TNBC in association with childbearing [3, 4, 43]. Our data show that *RRAS2* is overexpressed across ages but is more highly expressed in younger patients than in older ones. Within the younger age group, it is more highly expressed in parous women than in non-parous ones. Given that female mice overexpressing *RRAS2* develop BC if they have been breeding, *RRAS2* overexpression seems to be preferentially linked to BC developing during childbearing periods or a few years post-partum. Therefore, our data point to *RRAS2* as a driver oncogene, especially relevant to TNBC associated with childbearing.

Why *RRAS2* overexpression is especially associated with pregnancy-related breast cancers remains a question. We previously found that mice deficient in *Rras2* present defects in mammary gland development at puberty, with delayed branching and alveolar bud formation [12]. Conversely, we show here that mice overexpressing *RRAS2* produce significantly more alveolar buds during pregnancy than control ones and that mammary gland involution after weaning is significantly delayed. Together, our data suggest that R-RAS2 activity is important for the proliferation and remodeling of epithelial mammary tissue at different stages, and that its overexpression could drive the development of BC by promoting excessive cell proliferation. It has been proposed that post-lactation mammary gland involution may mimic aspects of wound healing, including the presence of activated fibroblasts, extracellular matrix deposition, and elevated matrix metalloproteinase levels, thus resembling a pro-tumorigenic wound environment [5]. In our *RRAS2*-overexpressing mouse models, we show there is increased cellularity in milk-producing alveoli and ducts during lactation in *RRAS2*-overexpressing females and that such increased cellularity persists weeks and months after parturition. Such an effect is detected as an increased number of proliferating epithelial cells in hyperplastic ductal epithelium. The altered breast involution in *RRAS2*-overexpressing mice is reminiscent of studies in humans suggesting that altered breast involution associated with childbearing not followed by breastfeeding could be a cause of the higher incidence of BC in postpartum women [43].

Although pregnancy and the postpartum period are linked, increasing evidence suggests the importance of differentiating breast cancer that occurs during pregnancy (Gestational BC) from BC that occurs during the postpartum period (Postpartum BC), which extends up to 5–10 years after childbirth depending on maternal age at delivery [5, 44–46]. The traditional definition of Pregnancy-associated breast cancer encompassed tumors identified during pregnancy or within the first few years after delivery, but this new classification is supported by their unique biological features and prognosis [6]. Large-cohort studies of Gestational BC have shown that clinical characteristics and prognosis of the patients are comparable to those observed in young, non-pregnant patients [47, 48]. However, Postpartum BC shows worse survival rates and double the risk of metastasis. Our two mouse lines overexpressing *RRAS2* displayed a notable occurrence of breast tumors among female breeders not currently pregnant or on lactation. This pattern could resemble cases of Postpartum BC, considering that BC emerging during pregnancy or lactation were in females that had had previous litters.

Although classic *RAS* genes are frequently mutated in human tumors, such mutations are uncommon in breast cancer [49, 50]. Additionally, although recent evidence has reported a role for overexpression of wild-type *KRAS*, *HRAS*, or *NRAS* in different carcinomas [51, 52], including invasive breast cancers [53], a driver role in their wild-type form for tumorigenesis in in vivo models has not yet been demonstrated. To our knowledge, our ubiquitous and tissue-specific mouse lines represent the first breast cancer mouse models driven by the overexpression of the wild-type, non-mutated version of a RAS small GTPase.

In addition to *RAS* family members, other genes commonly found to be mutated in other cancers, such as *PIK3CA*, are also found altered in some percentage of BC, having an impact on their characteristics and prognosis. A 44% of human breast cancers have been reported to bear mutations in *PIK3CA* (35.8% in cBioportal.org) of mainly Luminal A subtype [54]. Therefore, *PIK3CA* bearing activating mutations could be a driver oncogene in less than half of breast cancers. However, expression of catalytically active mutants of *PIK3CA* such as H1047R at physiological levels in mouse mammary tissue has failed to drive the development of breast cancer in the absence of accompanying mutations (reviewed in [55]). Therefore, to our knowledge, overexpressed wild-type *RRAS2* is the clearest driver gene leading to breast cancer without any additional intervention. Interestingly, another mouse model under study, which bears an activating Q72L mutation in *Rras2*, develops different types of cancer but not breast cancer [56], suggesting that the capacity of the wild-type R-RAS2 protein to cycle between the GTP-bound and the GDP-bound states might allow the emergence of tumors that otherwise would be locked if R-RAS2 is in the permanently active conformation.

We have identified breast cancer as the first human carcinoma that could emerge from the overexpression of wild-type *RRAS2*. Recently, we published that overexpression of wild-type *RRAS2* is also responsible for the development of chronic lymphocytic leukemia (CLL) [19]. Additionally, *RRAS2*-overexpressing mice develop cholangiocarcinomas (unpublished data). Besides CLL, breast cancer, and cholangiocarcinoma, considering the numerous human tumors that overexpress this gene [19], it is very likely that *RRAS2* overactivation through dose amplification will be implicated in many other cancers with significant impacts on human health. In this context, the association of the C allele at SNP rs8570 with higher *RRAS2* expression could become a useful tool to determine, based on allele frequency, which other human tumors might have *RRAS2* overexpression as an underlying driver mechanism.

## Conclusions

In conclusion, we show that the GTPase of the RAS-related subfamily R-RAS2 is overexpressed in the vast majority of human breast cancer samples regardless of grade, anatomic location or molecular type. However, *RRAS2* overexpression is even higher in triple-negative breast cancer and is associated with young age and parity. The human data suggest that overexpression of *RRAS2*, which does not carry activating mutations in the coding sequence, may be behind the development of breast cancer. Indeed, targeted overexpression of human *RRAS2* in mammary epithelial cells of genetically engineered mice provokes the development of triple-negative breast cancer in all female breeders, demonstrating a causal relationship between *RRAS2* overexpression and postpartum-associated breast cancer. In human patients, the highest levels of *RRAS2* expression are associated with shorter survival after diagnosis, higher proliferation rates and an overall poorer prognosis. The finding of a higher than normal frequency of the alternate C allele at SNP rs8570 position in the 3’UTR of *RRAS2*, the identification of somatic mutations at the SNP position, and the finding of gene amplification not only in breast tumor samples but also in blood, further support the idea that unmutated *RRAS2* overexpression is the most important driver gene in human breast cancer.

### Supplementary Information

Supplementary Material 1: Figure S1. a, Kaplan–Meier survival plot of breeding Rosa26-*RRAS2*^fl/fl^ x Sox2-Cre (*n* = 32), Rosa26-*RRAS2*^fl/fl^ x MMTV-Cre (*n* = 7), and Rosa26-*RRAS2*^fl/fl^ x Wap-Cre (*n* = 8) female mice allowed to age in the same housing conditions. The median survival of the Sox2-Cre group was 8.2 months, of the MMTV-Cre group was 6.6 months and that of the Wap-Cre group was 6.1 months. Significance was assessed with a long-rank Mantel-Cox test and no significant differences were detected. b, Immunoperoxidase staining of non-tumoral mouse tissues and tumoral tissue paraffin sections from Rosa26-*RRAS2*^fl/fl^ x Wap-Cre female mice showing estrogen receptor (ER), progesterone receptor (PR) and ErbB2 expression. Mouse endometrium was used as a positive control for ERa staining; mouse uterus as a positive control for PR staining and large bowel, colon, mucosa as a positive control for ErbB2 staining. Scale bar indicate 25 μm.

Supplementary Material 2: Figure S2. Box and whisker plots showing all the points, the median and mean ( +) values for relative *Rras2* mRNA expression in mammary gland epithelium of nulliparous 8 week-old C57BL/6 female mice (*n* = 4) receiving daily s.c. doses of β-estradiol, progesterone or just the vehicle for 5 consecutive days. Rras2 expression was measured by RT-qPCR and normalized to the expression of *Ctbp1* and *Gapdh*. Significance was assessed with a One-way ANOVA test. ns, *p* > 0.05.

Supplementary Material 3: Figure S3. a, Principal component analysis (PCA) showed the grouping of the 15 tumor samples, clearly differentiated from the 9 healthy tissue samples. b, Two tumor samples (CCM1 and CCW1: Table 2) were considered outliers and excluded from further analysis.

Supplementary Material 4: Figure S4. a, Two-dimensional plots of *RRAS2* and either *ESR1*, *PGR* or *ERBB2* normalized expression in all breast tumor samples from the TCGA database. Samples distribute in two clear groups of high (red) and low (blue) gene expression in the y-axis. b, Box and whisker plots showing the 10–90 percentile, the median and the mean ( +) values of normalized *RRAS2* mRNA expression data gathered from the TCGA database in human breast cancer samples classified as of estrogen receptor *ESR1*, progesterone receptor *PGR* and *ERBB2* low and high expressors according to their distribution in discrete cluster (panel a). Significance was assessed using a Mann–Whitney test.

Supplementary Material 5: Figure S5. Heatmap of unsupervised hierarchical clustering showing the top 0.5% of genes with the highest variance across mouse breast tumors developed under *RRAS2* overexpression and 1,116 human breast cancer samples from The Cancer Genome Atlas. A total of 70 genes met these criteria. For the heatmap, 80 random samples from each main molecular breast cancer subtype, along with the 15 mouse breast tumors, were selected. The values displayed correspond to the gene expression levels (normalized log2 pseudocounts). The molecular subtypes of human breast cancers are indicated by colored bars: blue for luminal A, green for luminal B, pink for HER2-enriched, red for TNBC, and yellow for mouse breast tumors.

Supplementary Material 6: Figure S6. Bar plot showing all the data points and the mean ± SEM of luciferase activity in lysates of the indicated human BC (BT549 and MCF7), T cell leukemic (Jurkat and HUT-78) and B cell CLL (MEC-1) cells transfected with the two reporter constructs shown in Fig. 7f, assessing significance with a two-sided unpaired t-test using Welch’s correction: **** *p* = 0.00004; ** *p* = 0.004; ns, not significant.

Supplementary Material 7: Figure S7. a, Number of tumors in the METABRIC study by their molecular type and age at diagnosis. b, Relative distribution of breast cancers, according to their molecular type, in the METABRIC study classified by age intervals. c, Mean ± s.e.m. of normalized *RRAS2* mRNA expression in BC samples of the METABRIC classified according by age at diagnosis and molecular type.

Supplementary Material 8.

Supplementary Material 9.

Supplementary Material 10.

## Data Availability

No datasets were generated or analysed during the current study.

## Abbreviations

BC: Breast cancer
TNBC: Triple-negative breast cancer
R-Ras2: RAS-related member 2
3’-UTR: 3 Untranslated region
SNP: Single-nucleotide polymorphism
Wap: Whey acidic protein
MMTV: Mouse mammary tumor virus
