# Supplementary figures and images for: Unmutated RRAS2 emerges as a key oncogene in post-partum-associated triple negative breast cancer
Source: Mol Cancer. 2024 Jul 10;23:142. doi: 10.1186/s12943-024-02054-3 (PMC11234613; doi:10.1186/s12943-024-02054-3)

a

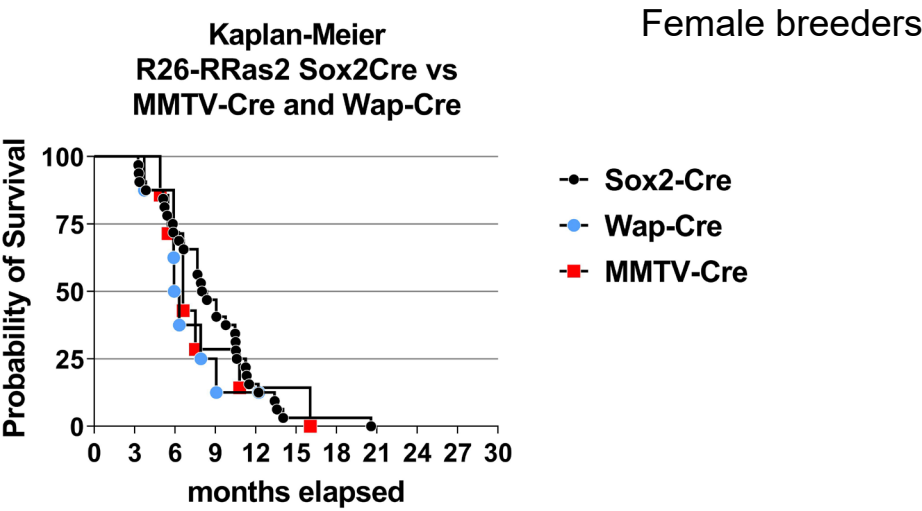

b

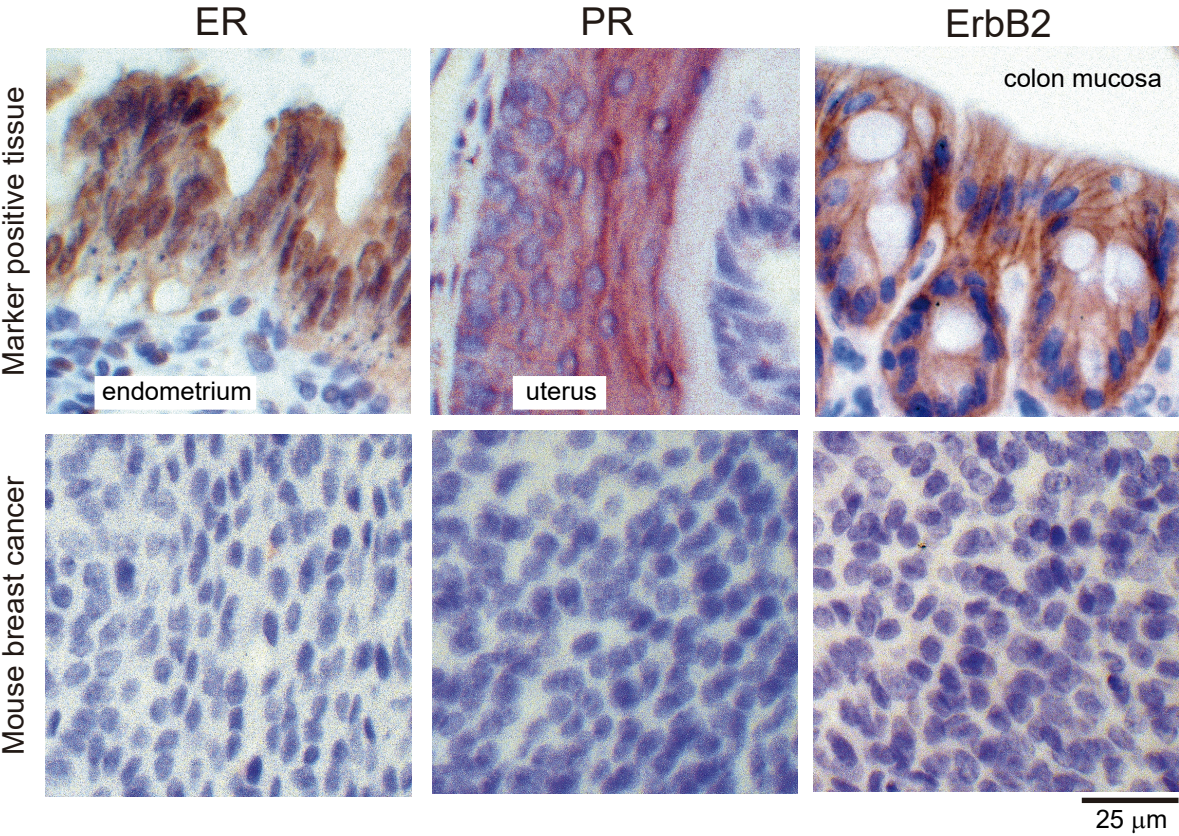

Figure S1

Supplement: Supplementary file 1 — Supplementary Material 1: Figure S1. a, Kaplan–Meier survival plot of breeding Rosa26-RRAS2fl/fl x Sox2-Cre (n = 32), Rosa26-RRAS2fl/fl x MMTV-Cre (n = 7), and Rosa26-RRAS2fl/fl x Wap-Cre (n = 8) female mice allowed to age in the same housing conditions. The median survival of the Sox2-Cre group was 8.2 months, of the MMTV-Cre group was 6.6 months and that of the Wap-Cre group was 6.1 months. Significance was assessed with a long-rank Mantel-Cox test and no significant differences were detected. b, Immunoperoxidase staining of non-tumoral mouse tissues and tumoral tissue paraffin sections from Rosa26-RRAS2fl/fl x Wap-Cre female mice showing estrogen receptor (ER), progesterone receptor (PR) and ErbB2 expression. Mouse endometrium was used as a positive control for ERa staining; mouse uterus as a positive control for PR staining and large bowel, colon, mucosa as a positive control for ErbB2 staining. Scale bar indicate 25 μm. [file 12943_2024_2054_MOESM1_ESM.pdf]

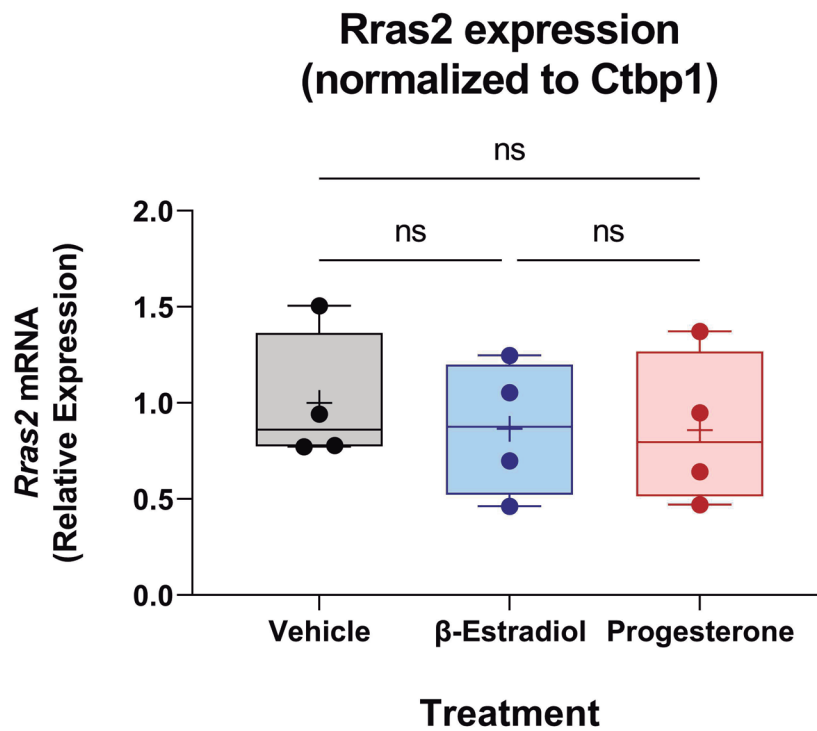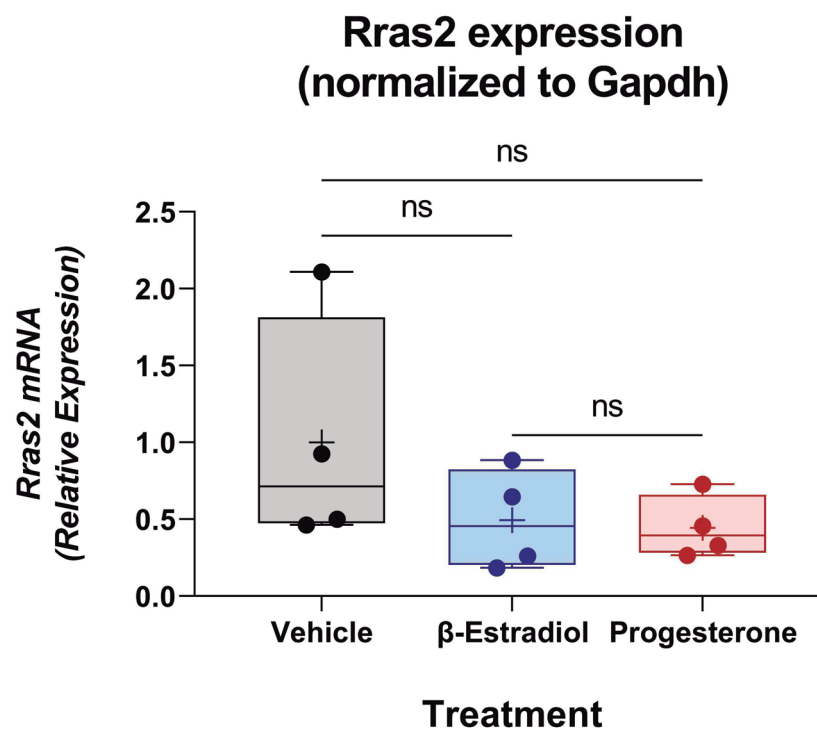

Figure S2

Supplement: Supplementary file 2 — Supplementary Material 2: Figure S2. Box and whisker plots showing all the points, the median and mean ( +) values for relative Rras2 mRNA expression in mammary gland epithelium of nulliparous 8 week-old C57BL/6 female mice (n = 4) receiving daily s.c. doses of β-estradiol, progesterone or just the vehicle for 5 consecutive days. Rras2 expression was measured by RT-qPCR and normalized to the expression of Ctbp1 and Gapdh. Significance was assessed with a One-way ANOVA test. ns, p > 0.05. [file 12943_2024_2054_MOESM2_ESM.pdf]

a

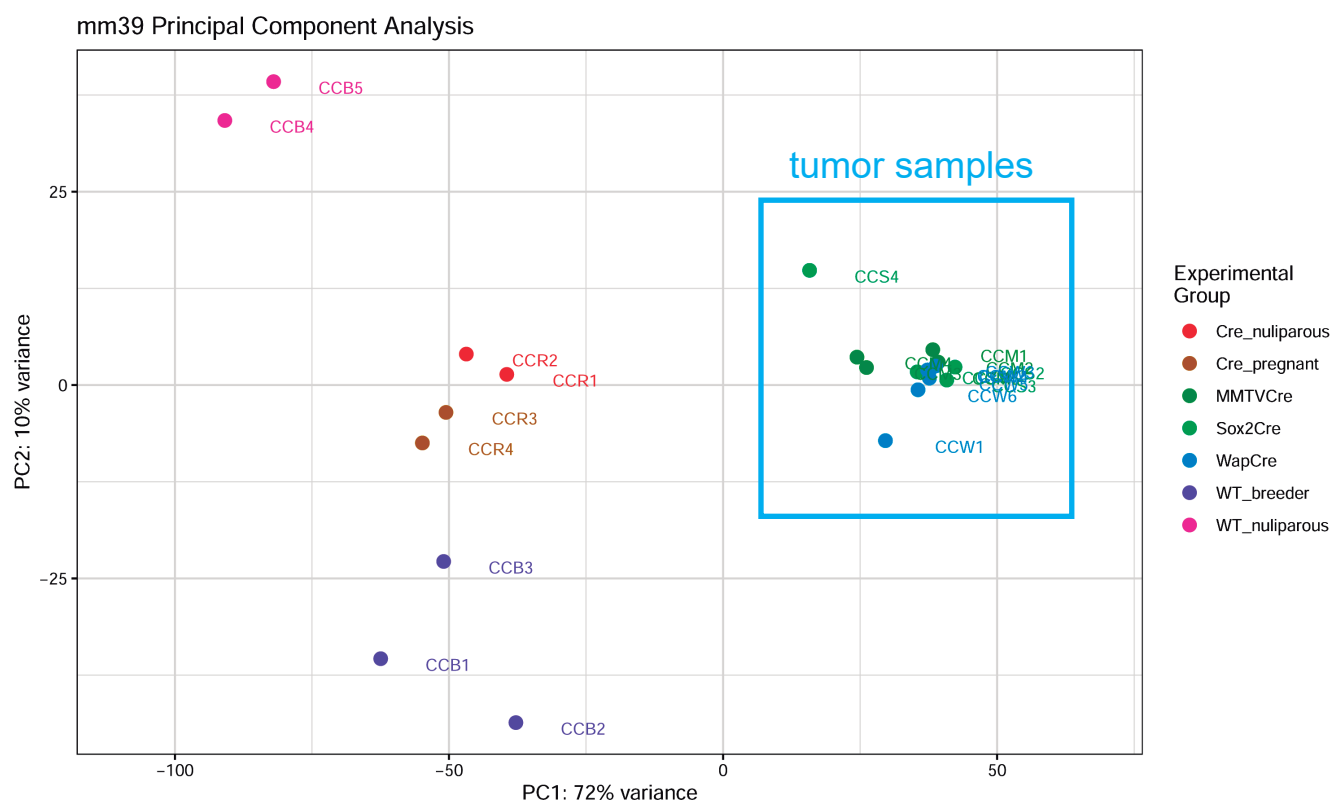

b

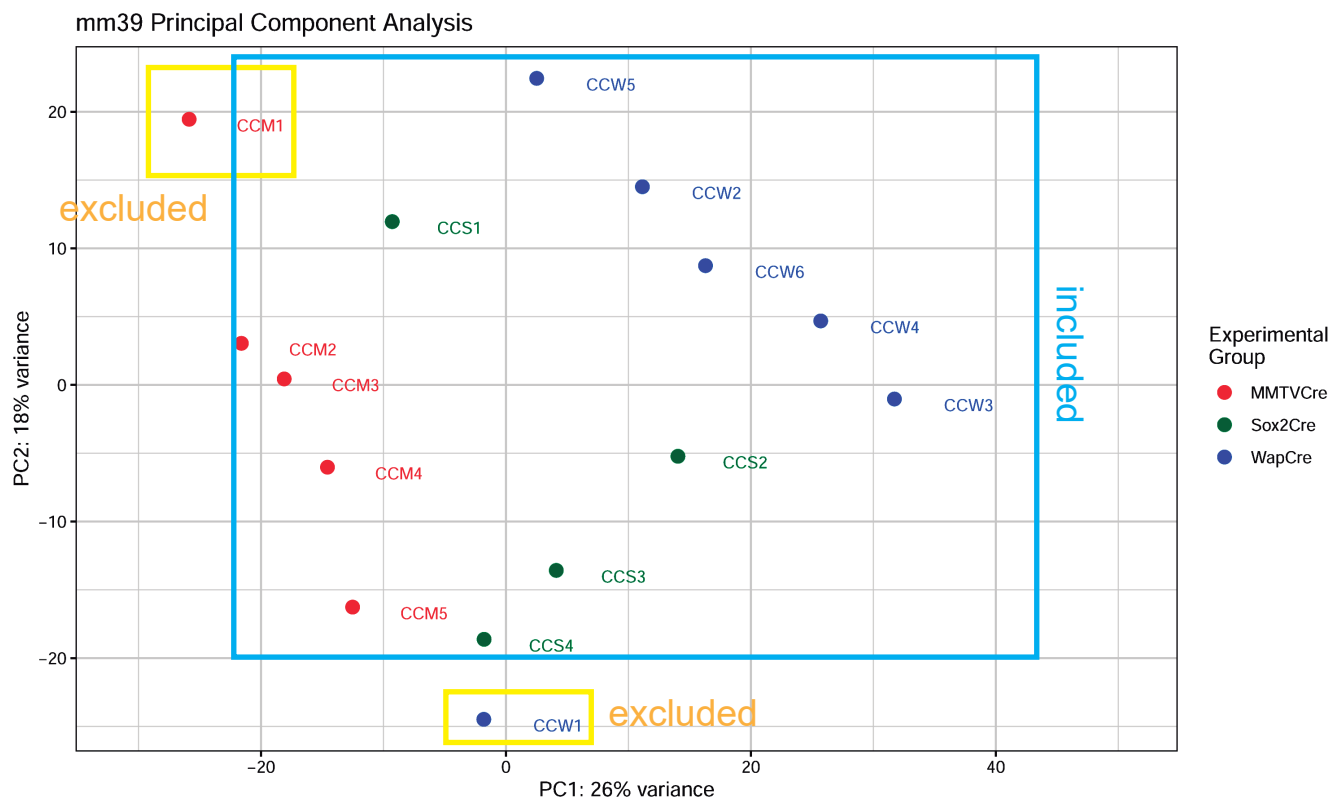

Figure S3

Supplement: Supplementary file 3 — Supplementary Material 3: Figure S3. a, Principal component analysis (PCA) showed the grouping of the 15 tumor samples, clearly differentiated from the 9 healthy tissue samples. b, Two tumor samples (CCM1 and CCW1: Table 2) were considered outliers and excluded from further analysis. [file 12943_2024_2054_MOESM3_ESM.pdf]

a

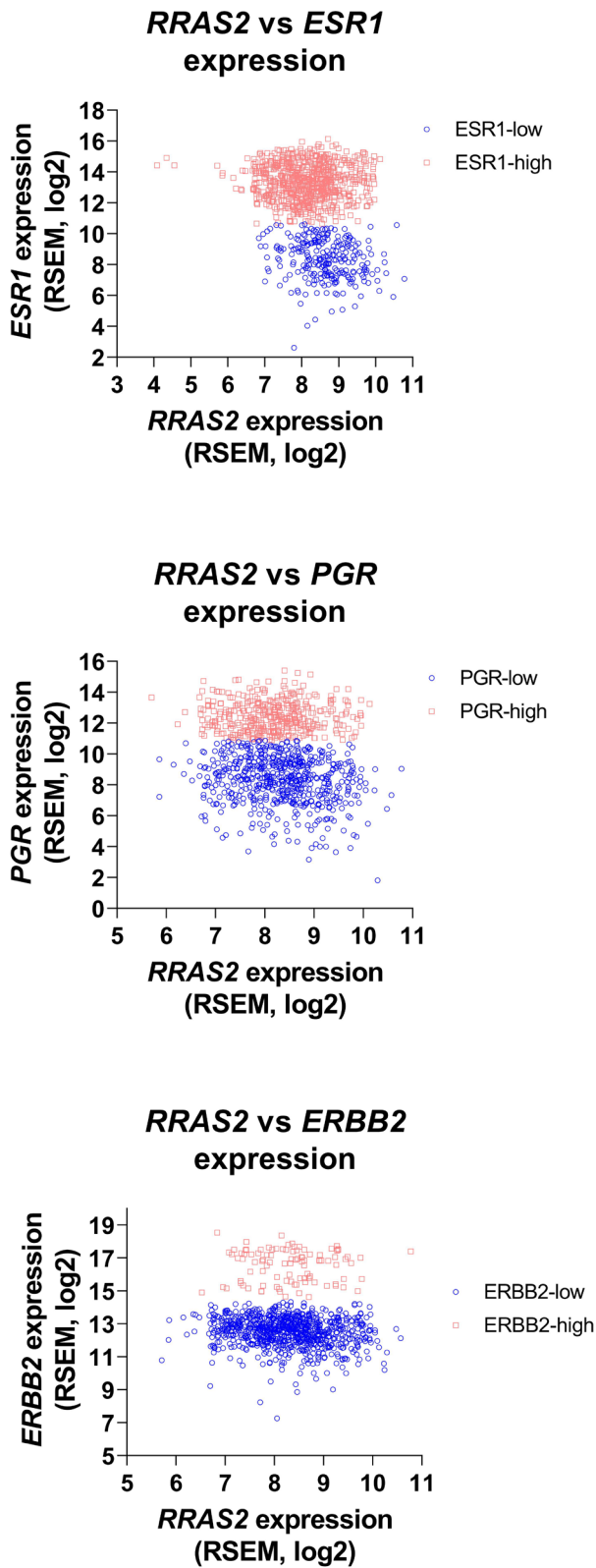

b

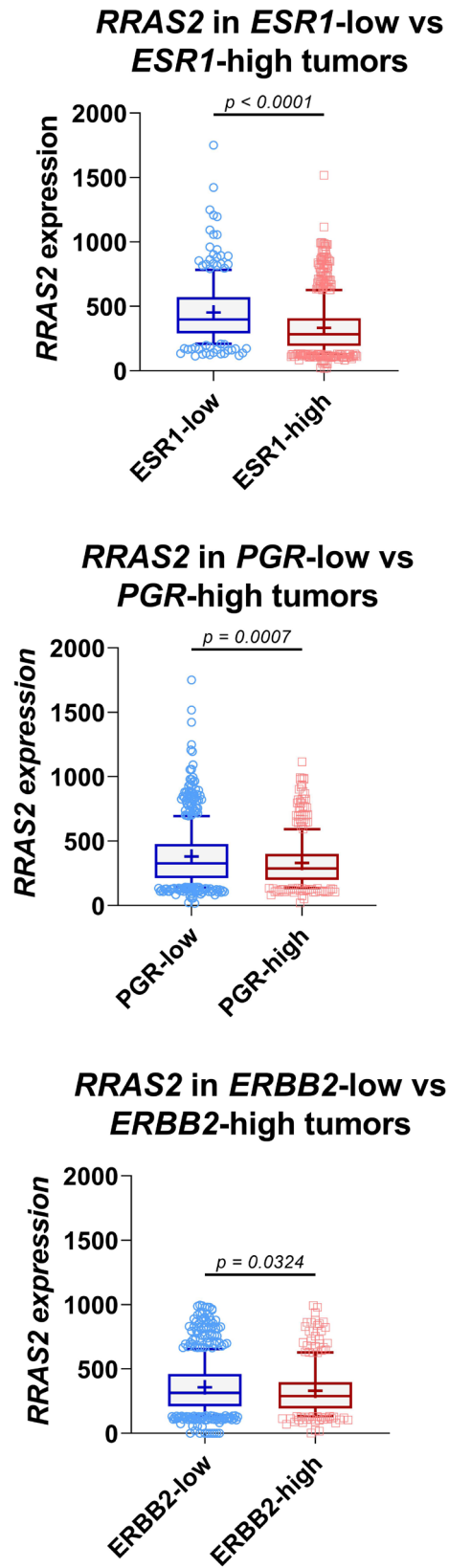

Figure S4

Supplement: Supplementary file 4 — Supplementary Material 4: Figure S4. a, Two-dimensional plots of RRAS2 and either ESR1, PGR or ERBB2 normalized expression in all breast tumor samples from the TCGA database. Samples distribute in two clear groups of high (red) and low (blue) gene expression in the y-axis. b, Box and whisker plots showing the 10–90 percentile, the median and the mean ( +) values of normalized RRAS2 mRNA expression data gathered from the TCGA database in human breast cancer samples classified as of estrogen receptor ESR1, progesterone receptor PGR and ERBB2 low and high expressors according to their distribution in discrete cluster (panel a). Significance was assessed using a Mann–Whitney test. [file 12943_2024_2054_MOESM4_ESM.pdf]

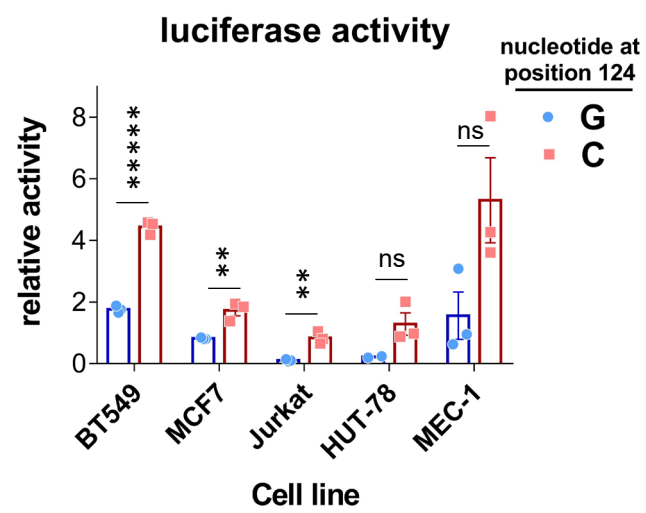

Figure S6

Supplement: Supplementary file 6 — Supplementary Material 6: Figure S6. Bar plot showing all the data points and the mean ± SEM of luciferase activity in lysates of the indicated human BC (BT549 and MCF7), T cell leukemic (Jurkat and HUT-78) and B cell CLL (MEC-1) cells transfected with the two reporter constructs shown in Fig. 7f, assessing significance with a two-sided unpaired t-test using Welch’s correction: **** p = 0.00004; ** p = 0.004; ns, not significant. [file 12943_2024_2054_MOESM6_ESM.pdf]
