# Supplementary material for: Unmutated RRAS2 emerges as a key oncogene in post-partum-associated triple negative breast cancer
Source: Mol Cancer. 2024 Jul 10;23:142. doi: 10.1186/s12943-024-02054-3 (PMC11234613; doi:10.1186/s12943-024-02054-3)
Supplement: Supplementary file 5 — Supplementary Material 5: Figure S5. Heatmap of unsupervised hierarchical clustering showing the top 0.5% of genes with the highest variance across mouse breast tumors developed under RRAS2 overexpression and 1,116 human breast cancer samples from The Cancer Genome Atlas. A total of 70 genes met these criteria. For the heatmap, 80 random samples from each main molecular breast cancer subtype, along with the 15 mouse breast tumors, were selected. The values displayed correspond to the gene expression levels (normalized log2 pseudocounts). The molecular subtypes of human breast cancers are indicated by colored bars: blue for luminal A, green for luminal B, pink for HER2-enriched, red for TNBC, and yellow for mouse breast tumors. [file 12943_2024_2054_MOESM5_ESM.pdf]

Heatmap of Gene Clustering in Murine RRAS2 versus Human Breast Cancer Samples by Molecular Subtype

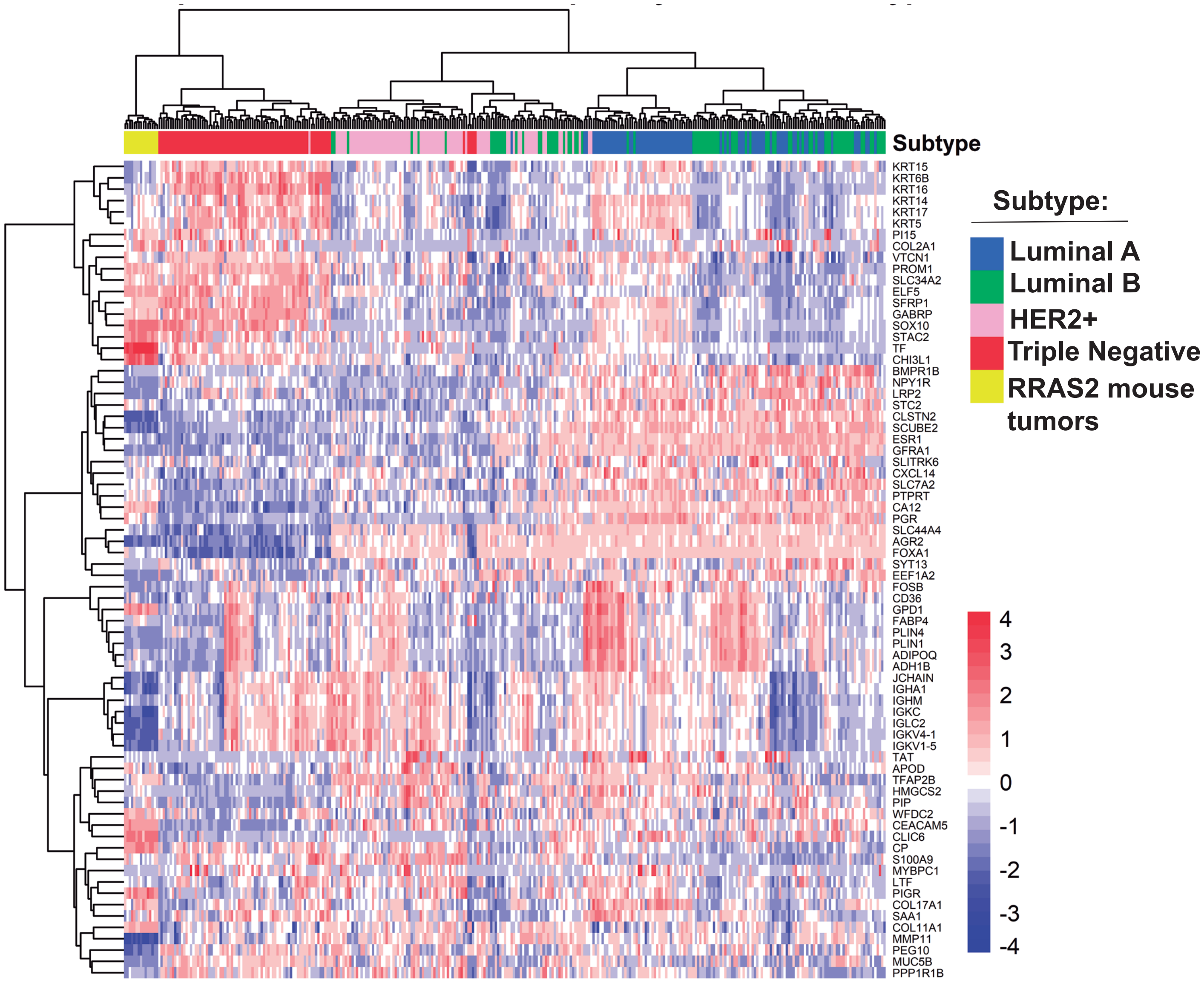

Figure S5
