# Supplementary material for: Unmutated RRAS2 emerges as a key oncogene in post-partum-associated triple negative breast cancer
Source: Mol Cancer. 2024 Jul 10;23:142. doi: 10.1186/s12943-024-02054-3 (PMC11234613; doi:10.1186/s12943-024-02054-3)
Supplement: Supplementary file 7 — Supplementary Material 7: Figure S7. a, Number of tumors in the METABRIC study by their molecular type and age at diagnosis. b, Relative distribution of breast cancers, according to their molecular type, in the METABRIC study classified by age intervals. c, Mean ± s.e.m. of normalized RRAS2 mRNA expression in BC samples of the METABRIC classified according by age at diagnosis and molecular type. [file 12943_2024_2054_MOESM7_ESM.pdf]

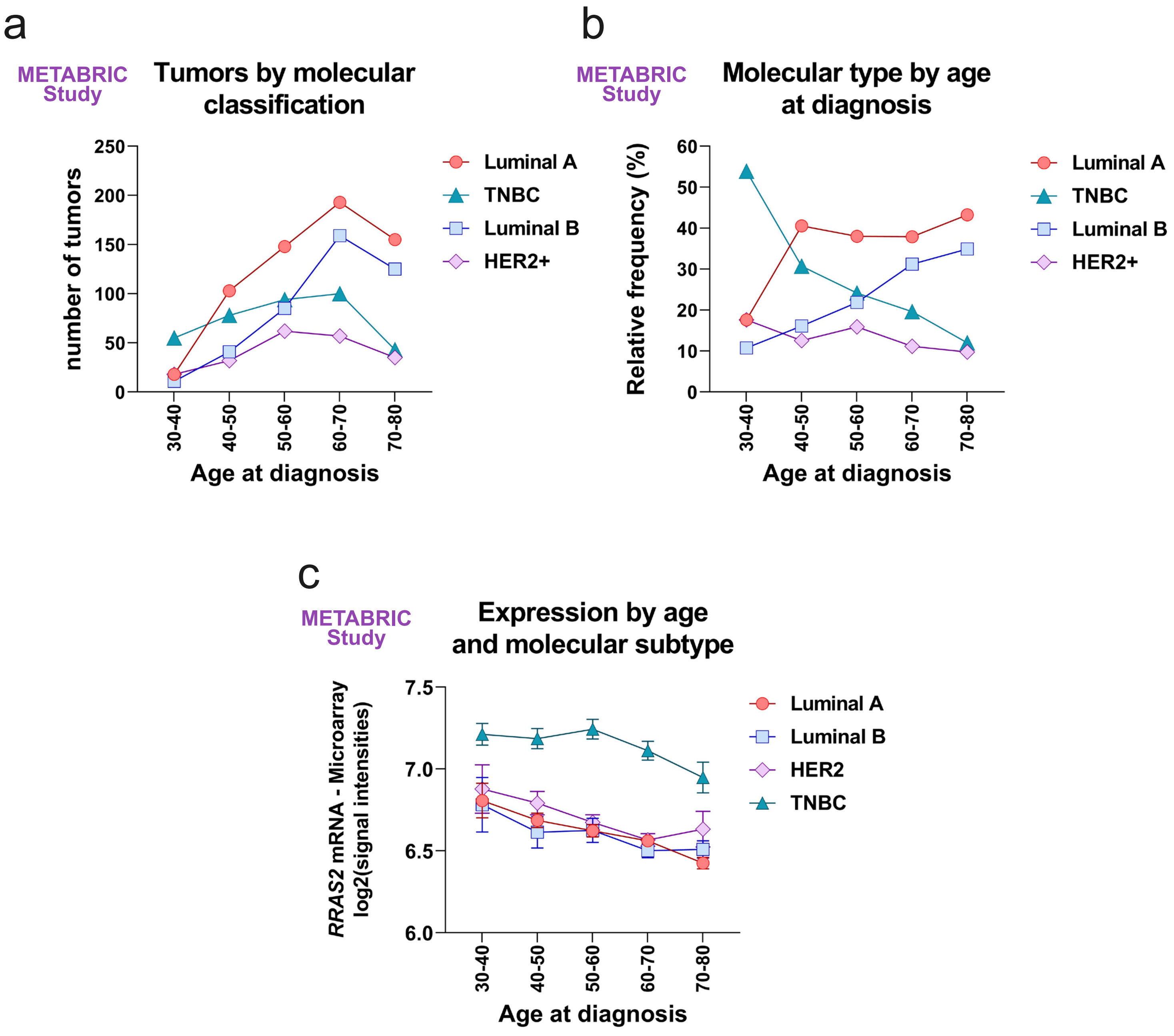
a
METABRIC Study
Tumors by molecular classification
number of tumors
250
200
150
100
50
0
30-40
40-50
50-60
60-70
70-80
Age at diagnosis
Luminal A
TNBC
Luminal B
HER2+
b
METABRIC Study
Molecular type by age at diagnosis
Relative frequency (%)
60
50
40
30
20
10
0
30-40
40-50
50-60
60-70
70-80
Age at diagnosis
Luminal A
TNBC
Luminal B
HER2+
c
METABRIC Study
Expression by age and molecular subtype
RRAS2 mRNA - Microarray log2(signal intensities)
7.5
7.0
6.5
6.0
30-40
40-50
50-60
60-70
70-80
Age at diagnosis
Luminal A
Luminal B
HER2
TNBC

Figure S7
